# Evidence of cryptic diversity in freshwater *Macrobrachium* prawns from Indochinese riverine systems revealed by DNA barcode, species delimitation and phylogenetic approaches

**DOI:** 10.1371/journal.pone.0252546

**Published:** 2021-06-02

**Authors:** Warut Siriwut, Ekgachai Jeratthitikul, Somsak Panha, Ratmanee Chanabun, Peng Bun Ngor, Chirasak Sutcharit

**Affiliations:** 1 Animal Systematics and Molecular Ecology Laboratory, Department of Biology, Faculty of Science, Mahidol University, Bangkok, Thailand; 2 Animal Systematics Research Unit, Department of Biology, Faculty of Science, Chulalongkorn University, Bangkok, Thailand; 3 Academy of Science, The Royal Society of Thailand, Dusit, Bangkok, Thailand; 4 Program in Animal Science, Faculty of Agricultural Technology, Sakon Nakhon Rajabhat University, Sakon Nakhon, Thailand; 5 Inland Fisheries Research and Development Institute (IFReDI), Fisheries Administration, Phnom Penh, Cambodia; 6 Wonders of the Mekong Project, Phnom Penh, Cambodia; National Cheng Kung University, TAIWAN

## Abstract

The diversity of Indochinese prawns in genus *Macrobrachium* is enormous due to the habitat diversification and broad tributary networks of two river basins: the Chao Phraya and the Mekong. Despite long-standing interest in SE-Asian decapod diversity, the subregional *Macrobrachium* fauna is still not yet comprehensively clarified in terms of taxonomic identification or genetic diversification. In this study, integrative taxonomic approaches including morphological examination, DNA barcoding, and molecular species delimitation were used to emphasize the broad scale systematics of *Macrobrachium* prawns in Indochina. Twenty-seven nominal species were successfully re-verified by traditional and molecular taxonomy. Barcode gap analysis supported broad overlapping of species boundaries. Taxonomic ambiguity of several deposited samples in the public database is related to inter- and intraspecific genetic divergence as indicated by BOLD discordance. Diagnostic nucleotide positions were found in six *Macrobrachium* species. Eighteen additional putative lineages are herein assigned using the consensus of species delimitation methods. Genetic divergence indicates the possible existence of cryptic species in four morphologically complex and wide-ranging species: *M*. *lanchesteri*, *M*. *niphanae*, *M*. *sintangense*, and some members of the *M*. *pilimanus* group. The geographical distribution of some species supports the connections and barriers attributed to paleo-historical events of SE-Asian rivers and land masses. Results of this study show explicitly the importance of freshwater ecosystems in Indochinese subregions, especially for the Mekong River Basin due to its high genetic diversity and species composition found throughout its tributaries.

## Introduction

DNA barcoding has been promoted as an effective molecular tool for rapid surveys of microbial [1, 2], floral [3], and faunal diversity [4]. Recently, DNA barcode data from single and multiple molecular loci have been integrated with various datasets and applied at broad and specific scales for uses such as food authentication and traceability [5, 6], wildlife forensics [7, 8], and ecological community [9] and systematic studies [10–12]. The selection of gene fragments from different sources of genomic DNA has been proposed to find a standard region for species identification within larger groups of organisms, such as chloroplast genes for plants [13], and mitochondrial genes for animals [14]. In the animal kingdom, the cytochrome C oxidase subunit I (COI) is widely used for species screening [14]. This mitochondrial gene contains a rich informative site, which is desirable for molecular systematic study. Moreover, additional markers for DNA barcode studies such as mitochondrial and nuclear ribosomal genes have been integrated with COI, expanding the power of species delimitation and improving sequence clustering results [15, 16].

The barcoding region has also shown significant utility in biodiversity studies that aim to rapidly identify unknown specimens collected from various habitat and sample types such as soil [17, 18], water [19], feces [20, 21], and dietary products [22, 23]. Moreover, it has been used to construct regional and local DNA barcode libraries for further biodiversity assessment [24, 25]. The use of integrative methods in systematics has shown remarkable ability to resolve species identification problems in organismal groups [26, 27]. Because they are cost-effective tools for species validation, classification, and phylogeography pattern testing, DNA taxonomy based on COI barcode, single-multi locus phylogeny, and species delimitation approaches have become popular in modern systematic research to clarify taxonomic problems, especially in morphologically complex groups [28–34]. As a consequence of applying integrative methods, cryptic diversity and phylogeographical patterns of organisms have been revised, especially in crustacean taxa [34–38]. Taxonomy of several freshwater and marine crustacean species has been refined by using the integration of morphological identification and molecular delimitations [39, 40].

The palaemonid prawn genus *Macrobrachium* Spence Bate, 1868 has economic importance worldwide. Recently, forty-nine *Macrobrachium* species have been promoted for fisheries industries [41]. These include widespread species such as *M*. *rosenbergii*, *M*. *lanchesteri*, and *M*. *sintangense*, which are consumed daily by local people as a protein resource. However, the high genetic diversity, evolutionary relationships and related biological adaptations of indigenous *Macrobrachium* species have only minimally been investigated in Asia; most research has focused on Neotropical American and Australian fauna [42–44]. In SE-Asia, the giant river prawn, *M*. *rosenbergii*, is the most economically important species [41], with the most advanced breeding program in the aquaculture industry [45]. Another study showed high genetic diversity of this species across drainages in SE-Asia [37]. Some life history traits of other Asian *Macrobrachium* species also have been tested using the integration of molecular, morphological, and ecological data [42, 46, 47].

The distribution of *Macrobrachium* prawns has been associated with river tributary networks across mainland SE-Asia including the Indochina subregion [48–50]. In addition, *Macrobrachium* prawns have been suggested for use in biological and ecological monitoring of Asian river ecosystems [51]. Some indigenous and endemic species are associated with specific habitats in this area such as *M*. *sirindhorn* and *M*. *spelaeus* from mountainous and limestone karst territory in Thailand; *M*. *lanatum* from southern Myanmar; and *M*. *dalatense*, *M*. *hungi*, and *M*. *saigonense*, which are endemic to the Mekong River basin in Cambodia and Vietnam [52–54].

The morphological and physiological changes during the developmental period of aquatic animals is assumed to be related to ecological factors such as current, water level and variation of habitat type [42, 55–58]. Habitat gradient, one of the selective mechanisms in aquatic animals, is thought to have had an impact on species diversification in several phylogenetic lineages of *Macrobrachium* that originated from the paleo ocean and further invaded freshwater habitats since the Mesozoic [59]. The adaptation to live as diadromous species offered high dispersal ability in *Macrobrachium* species (e.g., *M*. *nipponense*; see Chen, Shih [60]). The presence of environmental gradients in aquatic habitats plays a crucial role and may be a key for speciation in marine and freshwater animals [61]. The effect of altitude gradient on habitat diversification has been suggested in *Macrobrachium* diversity [62]. In Asia, some *Macrobrachium* species live in extreme environments, such as those which occupy subterranean water systems and exhibit troglobitic morphological features and life history adaptations [63–66].

Based on previous taxonomic records, up to forty-seven *Macrobrachium* species have been found throughout river basins of Indochina [48, 50, 52–54, 67–74]. The list of Indochinese *Macrobrachium* species and their type localities are given in Table 1. A group of widespread species such as *M*. *dienbienphuense*, *M*. *lanchesteri*, *M*. *niphanae*, *M*. *rosenbergii*, and *M*. *sintangense* were reported to occur together in riverine networks [50]. Some indigenous species were only found from a single locality, such as *M*. *chainatense*, *M*. *dolatum*, *M*. *spelaeus*, and *M*. *tratense*. Cambodia and Vietnam are less thoroughly explored than other countries in the region, although new species have recently been discovered from several remote areas, including one Cambodian and 12 Vietnamese taxa [52, 53, 73, 75–78]. These reports suggest the need for more integrative taxonomic assessments in the area.

**Table 1:** Species records of Indochinese *Macrobrachium* prawns.

Species	Type locality
1. *M*. *amplimanus* Cai and Dai, 1999	Mengla County, Xishuangbanna, southern China
2. *M*. *asperulum* (von Martens, 1868)	Shanghai, Eastern China
**3. *M*. *assamense* (Tiwari, 1955)**	Someswari River, near Siju, Garo Hill, Assam, India
4. *M*. *chainatense* Saengphan et al., 2019*	Mueang Chai Nat, Chai Nat, Thailand
5. *M*. *chilinhense* Dang, 2012	Chi Linh (Hai Duong), northern Vietnam
6. *M*. *dalatense* Nguyen, 2003	Mountain stream near the village of Krean, south of Dalat, south Vietnam
7. *M*. *dienbienphuense* Dang and Nguyen, 1972*	Dien Bien Phu, northern Vietnam
8. *M*. *dolatum* Cai et al., 2004	Trang, south Thailand
9. *M*. *duri* Wowor and Ng, 2010	Banten, Java, Indonesia
**10. *M*. *equidens* (Dana, 1852)**	Singapore
11. *M*. *eriocheirum* Dai, 1984*	Jingshan, Xishuangbanna, Yunnan, southern China
**12. *M*. *forcipatum* Ng, 1995**	Tasek Temengor, Perak, Malaysia
13. *M*. *hainanense* Parisi, 1919	Hainan Island, China
**14. *M*. *hendersoni* (De Man, 1906)**	Darjeeling, Western Bengal, India
15. *M*. *hirsutimanus* (Tiwari, 1952)*	Ban Pon, Nam Gae, north of Ban Sala, Nan, Thailand
16. *M*. *hungi* Nguyen, 2012	Tonlé Sap Lake, Cambodia
17. *M*. *idae* (Heller, 1862)	Borneo
18. *M*. *lanchesteri* (De Man, 1911)*	Songkla, southern Thailand
19. *M*. *lar* (Fabricius, 1793)	India
20. *M*. *latidactylus* (Thallwitz, 1891)	Sulawesi, Indonesia
**21. *M*. *malayanum* (Roux, 1934)**	Lasah, Plus valley, Perak, Malaysia
22. *M*. *mekongense* Dang, 1998	Mekong River, Dong Thap, southern Vietnam
23. *M*. *mieni* Dang, 1975	Hoa Binh, northern Vietnam
24. *M*. *naiyanetri* Siriwut, 2020*	Hui Prik, Cha-wang District, Nakhon Si Thammarat Province, Thailand.
**25. *M*. *neglectum* (De Man, 1905)**	Mergui Archipelago, Myanmar and northeastern Sumatra, Indonesia
26. *M*. *niphanae* Shokita and Takeda, 1989*	Nang Rong waterfall, Klong Yai and Khao Chamao, Thailand
**27. *M*. *nipponense* (De Haan, 1849)**	Japan
28. *M*. *palmopilosum* Siriwut, 2020*	Tat Man Waterfalls, Puea Sub-district, Chiang Klang District, Nan Province, Thailand
29. *M*. *phongnhaense* Do and Nguyen, 2014	Son Doong cave, Phong Nha-Ke Bang National Park, Quang Binh, Vietnam
30. *M*. *pilimanus* (De Man, 1879)	Moearalaboeh [Muara Labuh?], West Sumatra, Indonesia
31. *M*. *pilosum* Cai and Dai, 1999	Mountain stream near Mengban village, Mengla County, southern China
32. *M*. *puberimanus* Siriwut, 2020*	Mekong River at Wat Tha Khaek, Chiang Khan Sub-district, Chiang Khan District, Loei Province
**33. *M*. *rosenbergii* (De Man, 1879)**	Jakarta, Java, Indonesia
**34. *M*. *rogersi* (Tiwari, 1952)**	Arakan and Pegu Yomas, Burma
35. *M*. *saigonense* Nguyen, 2006	Near Hoa An bridge, Bien Hoa, northwest of Ho-Chi-Minh City, southern Vietnam
**36. *M*. *sintangense* (De Man, 1898)**	Sintang, Kapuas River, Borneo
**37. *M*. *sirindhorn* Naiyanetr, 2001**	Pong Nam Dung waterfall, Mae Soon, Fang, Chiang Mai, northern Thailand
38. *M*. *spelaeus* Cai and Vidthayanon, 2016*	Tham Phra Wangdaeng, Pitsanulok Province, northern Thailand
39. *M*. *suongae* Nguyen, 2003	Stream near An Phu village, South of Pleiku, central Vietnam
40. *M*. *superbum* (Heller, 1862)	Shanghai, eastern China
41. *M*. *suphanense* Saengphan et al., 2018*	Nikhom Krasiao, Dan Chang, Suphan Buri, Thailand
42. *M*. *thai* Cai et al., 2004*	Nong Khai, north-east Thailand
43. *M*. *tratense* Cai et al., 2004*	Khlong Fuai, Trat, eastern Thailand
44. *M*. *trompii* (De Man, 1898)	Borneo
45. *M*. *vietnamense* Dang and Nguyen, 1972	Ky Phu, Bac Thai, northern Vietnam
**46. *M*. *villosimanus* (Tiwari, 1949)**	Calcutta, India
**47. *M*. *yui* Holthuis, 1950**	Ninger, Puer County, Yunnan, southern China

Taxa in bold are representative species present in this study and “*” indicates species with topotype sample.

Because of the habitat diversity in Indochina and the insufficient data of taxonomic validity and diversity records, broad scale sampling of freshwater *Macrobrachium* prawns is crucially needed to fill gaps in the existing taxonomic data [48, 50]. Moreover, the decline of several prawn species and populations in their natural habitats is of critical concern because of habitat destruction [79, 80]. This study focuses on the improvement of regional data through integrative taxonomy, emphasizing species identification, species delineation, phylogenetic relationships and geographical distributions. In this study, newly determined COI sequences of twenty-three nominal species of Indochinese prawns are provided. The re-collection of topotypes of some described taxa is used to reconfirm species identity and position within the phylogenetic tree. The representative taxa used in this study cover 53% of all nominal taxa recorded in the Indochina subregion (Table 1). The results of this study can benefit the conservation management of Indochinese freshwater prawns.

## Methods

### Specimen collection, preparation and identification

**Fig 1:**
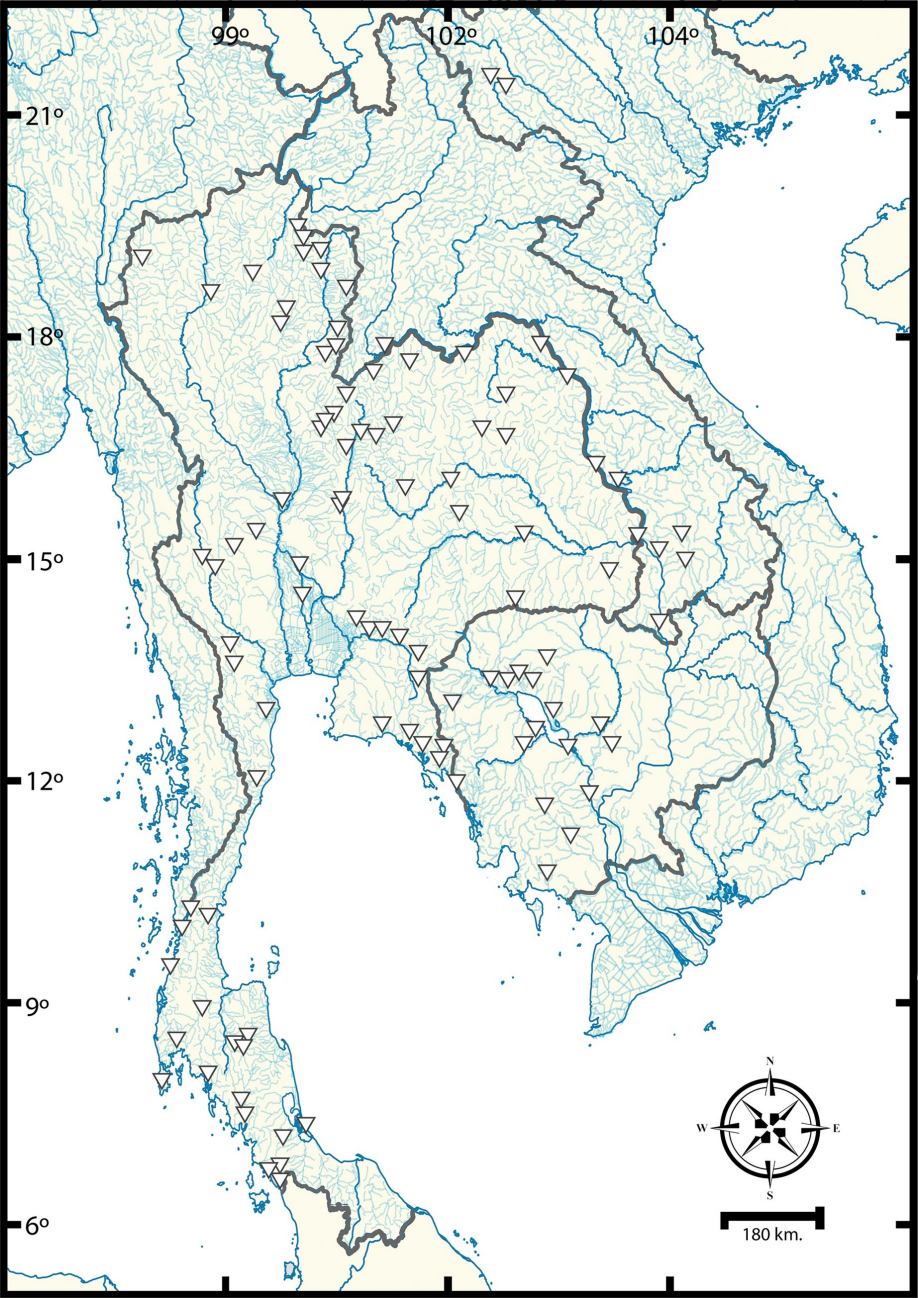
Sampling localities of *Macrobrachium* in the Indochinese subregion.

Prawns were collected from natural habitats during 2017–2019 in various river systems of Indochinese countries, including Thailand, Laos, Cambodia, and Vietnam (Fig 1). Collecting permission in remote areas of Thailand was granted by the Department of National Parks, Wildlife and Plant Conservation, Thailand (DNP 0907.4/14262). Permission in Cambodia was granted by the Fisheries Administration Ministry of Agriculture, Forestry and Fisheries of Cambodia. Animal use in this project strictly followed the recommended protocols approved by Chulalongkorn University (Protocol Review No. 1723018) and Mahidol University’s Institute Animal Care and Use Committee (MU-IACUC) under approval number MU-IACUC 2018/004.

Prawns were trapped by using artificial baited net traps and by net sieving. GPS coordinates were recorded in each collecting locality. The collecting localities were illustrated in geographical map via QGIS v3.18 [81]. The template of hydrological basin was extracted from FAO GeoNetwork opensource database [82]. Some of the collected specimens were photographed for their live habitus coloration in a small aquarium with substrate and background. Specimens were euthanized by the two-step method following AVMA Guidelines for the Euthanasia of Animals [83]. Prawns were anesthetized by the gradual addition of 95% (v/v) ethanol to the container, starting from approximately 5% (v/v) concentration. Later, the fully anesthetized specimens were persevered with 95% (v/v) ethyl alcohol for further morphological and molecular analyses. Morphological identification was made based on taxonomic literature of SE-Asian fauna as follows: Cai, Naiyanetr [48], Hanamura, Imai [50], Xuan [52], Saengphan, Panijpan [69], Saengphan, Panijpan [70], Cai and Dai [84], Holthuis [85], Wowor and Short [86], Cai and Vidthayanon [87], Siriwut, Jeratthitikul [88].

### DNA extraction, amplification, and sequencing

Prawn specimens were dissected to obtain somatic tissue. The Nucleospin^TM^ DNA extraction kit (MACHEREY-NAGEL, Germany) was used for genomic DNA extraction. Genomic DNA yield was quantified by Nanodrop spectrophotometer (Thermo Scientific, USA). Cytochrome C oxidase subunit I (COI) gene was amplified using universal LCO1490 forward primer [14, 89] and a newly designed reverse primer specific for *Macrobrachium*, MacroR (5’-GCGGGTAGRATTAARATRTATACTTC-3’). The standard PCR mixture contained 1 μl of genomic DNA, 2.5 μl of forward and reverse primers, 25 μl of EmeraldAmp PCR Maser Mix (TAKARA BIO, Japan) and 18 μl of ddH_2_O.

PCR was carried out by an Eppendorf Master Cycler Pro S (Eppendorf, Germany) with gradient temperature function. The PCR conditions were set as follows: 94°C for 5 min as an initial step followed by 36 cycles of 94°C for 30 s for denaturation, 41–45°C for 40 s, 72°C for 15 s for extension, and then final extension at 72°C for 10 min. The amplicon products were run by 1% agarose gel electrophoresis stained with SYBR Safe illuminant (Invitrogen, USA). Observation was made under a UV gel documentation machine. Later, the target products were purified using a QIAquick purification kit (QIAGEN, Germany). The purified products were sequenced by commercial sequencing company (Macrogen and Bioneer, Korea) using Applied Biosystems automatic sequencer.

### Sequence editing, alignment, and phylogenetic reconstruction

Prawn COI gene sequences were aligned with deposited sequences in the GenBank library using the BLASTn algorithm to verify the correct group of organisms from obtained sequences. The verified sequences were assembled, edited, and aligned using MUSCLE algorithm [90] in MEGA 7 [91]. Input files for each phylogenetic method were configured in MEGA 7 and Mesquite v3.61 [92]. The best fit nucleotide substitution model was sampled by using JModelTest v2.1.10 [93]. In this study, maximum likelihood (ML) and Bayesian inference (BI) methods were applied to reconstruct phylogenetic trees from the COI dataset. For ML analysis, the dataset was analyzed in RAxML 8.0.0v [94] via CIPRES portal [95] and W-IQ-TREE [96] via online server (http://iqtree.cibiv.univie.ac.at/) with 1,000 bootstrapping tests and default parameter settings. The Maximum likelihood phylogenetic tree based on RAxML program was constructed under GTR+CAT model for the best-fit nucleotide substitution. Bayesian inference was conducted in MrBayes, ver. 3.2.6. [97]. Ten million MCMC generations were sampled and the burn-in fragment discarding was configured as 0.5. The consensus tree as implemented from 50% majority rules was harvested at the final stage, and the raw tree topology file was then illustrated in FigTree [98]. Clade support was defined as monophyly when support values exceeded the accepted threshold of the bootstrap values 70% for RaxML and 90% for W-IQ-TREE, and 0.95 of posterior probabilities for BI. A p-distance of nucleotide comparison was calculated in MEGA 7. The number of nucleotide differences and genetic distance were compared for both inter–and intraspecific variation. A bar chart depicting DNA barcode gaps was constructed in Excel and re-drawn in Adobe Illustrator.

### DNA barcode analysis

Sequence datasets were registered and deposited in BOLD system [99]. The taxonomic account, voucher specimen ID, collecting locality, and voucher depositor were incorporated into the system for further analysis. Available barcoding sequences from previous literature such as Hanamura, Imai [50], Saengphan, Panijpan [69], Saengphan, Panijpan [70], Siriwut, Jeratthitikul [88], Wowor, Muthu [100] were included in the dataset. A list of sequences used in barcode analysis is presented in Table 2. The analysis tools in BOLD were used to calculate nucleotide diversity, barcoding gap, diagnosis nucleotide, and sequence cluster. All sequences in the dataset were initially registered to obtain Process ID and to check for possible pseudogenes or nuclear copies of mitochondrial DNA (NUMTs) based on the occurrence of stop codons or frameshift mutations in each submitted sequence. The genetic distances among sequences was analyzed using the MUSCLE option in the distance summary tool of the BOLD workbench. The barcode gap analysis was performed to indicate the genetic distance distribution between the operational targeted species and the nearest neighbor species. Two distance models were used: pairwise distance and K2-P distance. The alignment of sequence datasets was carried out under the MUSCLE option. The diagnostic nucleotide of non-singleton species was predicted under K2-P distance model and MUSCLE alignment algorithm. All parameters were set as default. According to a barcode index number (BIN) automatically assigned [101] in the BOLD system, the sequence dataset was checked with BIN records. The clustering result using the Refined Single Linkage algorithm (RESL) initially validated the putative OTUs in the sequence dataset. Alternatively, the DNA barcode gap plot was constructed via MEGA under the distance calculation panel; the dataset obtained from pairwise comparison, intraspecific and interspecific distances of defined sequences was comparatively implemented to construct a barcode gap diagram.

**Table 2:** *Macrobrachium* species samples used in this study including previously deposited sequences from BOLD.

Operational taxonomic unit ID	BOLD Process ID	BIN	Catalog No/GenBank ID	Locality	GPS coordinates
*M*. *chainatense*	PRSEA144-20	BOLD:AEC8313	CUMZ_MP00161	Mueang, ‎Chai Nat, Thailand	15° 13’ 19.8192"N, 100° 6’ 6.1158"E
	PRSEA145-20	BOLD:AEC7811	CUMZ_MP00162	Mueang, ‎Chai Nat, Thailand	15° 13’ 19.8192"N, 100° 6’ 6.1158"E
*M*. *dienbienphuense*	PRSEA011-20	BOLD:AEC8122	CUMZ_MP00028	Nam Nao, Phetchabun, Thailand	16° 39’ 2.88"N, 101° 46’ 22.8354"E
	PRSEA015-20	BOLD:AEC7844	CUMZ_MP00032	Wang Thong, Phitsanulok, Thailand	16° 49’ 20.6364"N, 100° 25’ 56.5392"E
	PRSEA026-20	BOLD:AEC7844	CUMZ_MP00043	Pong, Phayao, Thailand	19°07’11.1”N, 100°16’49.8"E
	PRSEA044-20	BOLD:AEC6883	CUMZ_MP00061	Mueang, Sa Kaeo, Thailand	13°48’33.6”N, 102°03’16.8"E
	PRSEA045-20	BOLD:AEC9528	CUMZ_MP00062	Mueang, Nakhon Nayok, Thailand	14°11’46.1”N, 101°11’36.5"E
	PRSEA051-20	BOLD:AEC8122	CUMZ_MP00068	Det Udom, Ubon Ratchathani, Thailand	14°26’46.2”N, 105°07’16.1"E
	PRSEA052-20	BOLD:AEC6883	CUMZ_MP00069	Mueang, Sa Kaeo, Thailand	13°48’33.6”N, 102°03’16.8"E
	PRSEA053-20	BOLD:AEC6883	CUMZ_MP00070	Mueang, Trat, Thailand	12°19’30.3”N, 102°30’01.0"E
	PRSEA055-20	BOLD:AEC8122	CUMZ_MP00072	Mueang, ‎Chaiyaphum, Thailand	15°56’27.7”N, 102°01’22.2"E
	PRSEA058-20	BOLD:AEC6883	CUMZ_MP00075	Lan Saka, Nakhon Si Thammarat, Thailand	8°20’56.8”N, 99°47’47.5"E
	PRSEA063-20	BOLD:AEC8122	CUMZ_MP00080	Wang Sam Mo, Udon Thani, Thailand	16°56’52.2”N, 103°28’24.8"E
	PRSEA070-20	BOLD:AEC8122	CUMZ_MP00087	Chiang Kham, Phayao, Thailand	19°33’41.7”N, 100°17’42.0"E
	PRSEA078-20	BOLD:AEC9528	CUMZ_MP00095	Bueng Sam Phan, Phetchabun, Thailand	15°49’52.5”N, 101°02’08.0"E
	PRSEA091-20	BOLD:AEC8122	CUMZ_MP00108	Phu Kradueng, Loei, Thailand	16°51’40.0”N, 101°54’29.6"E
	PRSEA116-20	BOLD:AEC6883	CUMZ_MP00133	Soi Dao, Chanthaburi, Thailand	13°07’12.3”N, 102°13’02.6"E
	PRSEA131-20	BOLD:AEC8122	CUMZ_MP00148	Det Udom, Ubon Ratchathani, Thailand	14°26’46.2”N, 105°07’16.1"E
	PRSEA132-20	BOLD:AEC8122	CUMZ_MP00149	Det Udom, Ubon Ratchathani, Thailand	14°26’46.2”N, 105°07’16.1"E
	PRSEA133-20	BOLD:AEC8122	CUMZ_MP00150	Det Udom, Ubon Ratchathani, Thailand	14°26’46.2”N, 105°07’16.1"E
	PRSEA141-20	BOLD:AEC7845	CUMZ_MP00158	Noen Maprang, Phitsanulok, Thailand	16°41’46.8”N, 100°39’24.5"E
	PRSEA150-20	BOLD:AEC6883	CUMZ_MP00167	Banteay Srei, Siem Reap, Cambodia	13°35’43.1”N, 103°57’42.6"E
	PRSEA152-20	BOLD:AEC8122	CUMZ_MP00169	Krolanh, Siem Reap, Cambodia	13°35’29.7”N, 103°24’16.3"E
	PRSEA153-20	BOLD:AEC6883	CUMZ_MP00170	Thlea Ma Om, Pursat, Cambodia	12°31’34.7”N, 104°03’16.2"E
	PRSEA154-20	BOLD:AEC6883	CUMZ_MP00171	Kralanh River, Siem Reap, Cambodia	13°35’29.7”N, 103°24’16.3"E
	PRSEA162-20	BOLD:AEC6883	CUMZ_MP00179	Banteay Srei, Siem Reap, Cambodia	13°35’43.1”N, 103°57’42.6"E
	PRSEA175-20	BOLD:AEC8121	CUMZ_MP00192	Nam Nua River, Dien Bien Phu, Vietnam	21°13’11.1”N, 103°03’10.6"E
*M*. *equidens*	GBCMD2468-09		FM958063	Khatib Bongsu, Singapore*	
*M*. *forcipatum*	PRSEA113-20	BOLD:AED0129	CUMZ_MP00130	Kathu, Phuket, Thailand*	
	PRSEA114-20	BOLD:AEC7831	CUMZ_MP00131	Mueang, Phangnga, Thailand*	
*M*. *hendersoni*	PRSEA118-20	BOLD:AEC6742	CUMZ_MP00135	Si Sawat, Kanchanaburi, Thailand	14°23’04.3”N, 99°08’20.0"E
	PRSEA119-20	BOLD:AEC6742	CUMZ_MP00136	Si Sawat, Kanchanaburi, Thailand	14°23’04.3”N, 99°08’20.0"E
	PRSEA130-20	BOLD:AEC6742	CUMZ_MP00147	Dan Makham Tia, Kanchanaburi, Thailand	13°50’42.9”N, 99°23’59.3"E
*M*. *hirsutimanus*	PRSEA014-20	BOLD:AED0116	CUMZ_MP00031	Wang Thong, Phitsanulok, Thailand*	
	PRSEA031-20	BOLD:AED0116	CUMZ_MP00048	Wang Thong, Phitsanulok, Thailand*	
	PRSEA046-20	BOLD:AED0116	CUMZ_MP00063	Suan Phueng, Ratchaburi, Thailand*	
	PRSEA048-20	BOLD:AED0116	CUMZ_MP00065	Tha Yang, Phetchaburi, Thailand*	
	PRSEA049-20	BOLD:AED0116	CUMZ_MP00066	Mueang, Nakhon Nayok, Thailand*	
	PRSEA050-20	BOLD:AED0116	CUMZ_MP00067	Bo Rai, Trat, Thailand*	
	PRSEA054-20	BOLD:AED0116	CUMZ_MP00071	Mueang, Nakhon Nayok, Thailand*	
	PRSEA077-20	BOLD:AED0116	CUMZ_MP00094	Noen Maprang, Phitsanulok, Thailand*	
	PRSEA122-20	BOLD:AED0116	CUMZ_MP00139	Wang Thong, Phitsanulok, Thailand*	
*M*. *lanatum*	GBCMD2450-09	BOLD:AAX4841	FM958081	Bengkulu, Sumatra*	
*M*. *lanchesteri*	PRSEA001-20	BOLD:AAJ1427	CUMZ_MP00018	Wang Thong, Phitsanulok, Thailand	16°49’20.3”N, 100°25’51.9"E
	PRSEA002-20	BOLD:AAJ1427	CUMZ_MP00019	Wiang Chai, Chiang Rai, Thailand	19°52’10.7”N, 99°56’42.8"E
	PRSEA004-20	BOLD:AAJ1427	CUMZ_MP00021	Pong, Phayao, Thailand	19°09’25.3”N, 100°16’52.9"E
	PRSEA016-20	BOLD:AAJ1427	CUMZ_MP00033	Chiang Klang, Nan, Thailand	19°17’13.8”N, 100°51’24.6"E
	PRSEA024-20	BOLD:AAJ1427	CUMZ_MP00041	Ban Khok, Uttaradit, Thailand	18°05’38.9”N, 101°07’20.8"E
	PRSEA025-20	BOLD:AAJ1427	CUMZ_MP00042	Ban Khok, Uttaradit, Thailand	18°05’38.9”N, 101°07’20.8"E
	PRSEA042-20	BOLD:AAJ1427	CUMZ_MP00059	Phu Kradueng, Loei, Thailand	16°51’40.0”N, 101°54’29.6"E
	PRSEA066-20	BOLD:AAJ1427	CUMZ_MP00083	Mueang, Nakhon Sawan, Thailand	15°37’16.9”N, 100°05’37.5"E
	PRSEA072-20	BOLD:AAJ1427	CUMZ_MP00089	Sathing Phra, Songkhla, Thailand	7°25’01.4”N, 100°25’04.0"E
	PRSEA073-20	BOLD:AAJ1427	CUMZ_MP00090	Sathing Phra, Songkhla, Thailand	7°25’01.4”N, 100°25’04.0"E
	PRSEA074-20	BOLD:AAJ1427	CUMZ_MP00091	Wichian Buri, Phetchabun, Thailand	15°34’45.5”N, 101°04’49.6"E
	PRSEA076-20	BOLD:AAJ1427	CUMZ_MP00093	La-un, Ranong, Thailand	10°06’37.1”N, 98°45’32.5"E
	PRSEA123-20	BOLD:AAJ1427	CUMZ_MP00140	Kabin Buri, Prachin Buri, Thailand	13°56’14.8”N, 101°55’19.1"E
	PRSEA124-20	BOLD:AAJ1427	CUMZ_MP00141	Mueang, Kalasin, Thailand	16°27’53.4”N, 103°30’17.7"E
	PRSEA125-20	BOLD:AAJ1427	CUMZ_MP00142	Sawang Daen Din, Sakon Nakhon, Thailand	17°28’35.9”N, 103°28’28.8"E
	PRSEA147-20	BOLD:AAJ1427	CUMZ_MP00164	Preah Netr Preah, Banteay Meanchey, Cambodia	13°37’20.2”N, 103°11’51.9"E
	PRSEA148-20	BOLD:AAJ1427	CUMZ_MP00165	Preah Netr Preah, Banteay Meanchey, Cambodia	13°37’20.2”N, 103°11’51.9"E
	PRSEA149-20	BOLD:AAJ1427	CUMZ_MP00166	Preah Netr Preah, Banteay Meanchey, Cambodia	13°37’20.2”N, 103°11’51.9"E
	PRSEA156-20	BOLD:AAJ1427	CUMZ_MP00173	Puok, Siem Reap, Cambodia	13°27’16.7”N, 103°44’24.0"E
	PRSEA157-20	BOLD:AAJ1427	CUMZ_MP00174	Stoung, Kampong Thom, Cambodia	12°56’41.5”N, 104°34’57.9"E
	PRSEA197-20	BOLD:AAJ1427	CUMZ_MP00214	Pakse, Champasak, Laos	15°06’50.7”N, 105°48’49.2"E
	PRSEA198-20	BOLD:AAJ1427	CUMZ_MP00215	Pakse, Champasak, Laos	15°06’50.7”N, 105°48’49.2"E
*M*. *latidactylus*	PRSEA088-20	BOLD:ACS9506	CUMZ_MP00105	Mueang, Krabi, Thailand	8°04’48.9”N, 98°55’08.5"E
	PRSEA108-20	BOLD:ACS9506	CUMZ_MP00125	La-ngu, Satun, Thailand	6°54’22.3”N, 99°48’42.2"E
*M*. *malayanum*	PRSEA139-20	BOLD:AEC7359	CUMZ_MP00156	Wang Wiset, Trang, Thailand*	
	PRSEA134-20	BOLD:AEC7359	CUMZ_MP00151	Wang Wiset, Trang, Thailand*	
	PRSEA140-20	BOLD:AEC7359	CUMZ_MP00157	Wang Wiset, Trang, Thailand*	
*M*. *naiyanetri*	PRSEA013-20	BOLD:AEC9525	CUMZ_MP00030	Mueang, Phetchabun, Thailand*	
	PRSEA089-20	BOLD:AEC9525	CUMZ_MP00106	Khlung, Chanthaburi, Thailand*	
	PRSEA111-20	BOLD:AEC9526	CUMZ_MP00128	Chawang, Nakhon Si Thammarat, Thailand*	
	PRSEA112-20		CUMZ_MP00129	Chawang, Nakhon Si Thammarat, Thailand*	
	PRSEA117-20	BOLD:AEC9523	CUMZ_MP00134	Rattaphum, Songkhla, Thailand*	
	PRSEA135-20	BOLD:AEC9526	CUMZ_MP00152	Chawang, Nakhon Si Thammarat, Thailand*	
	PRSEA136-20	BOLD:AEC9526	CUMZ_MP00153	Chawang, Nakhon Si Thammarat, Thailand*	
	PRSEA151-20	BOLD:AEC9524	CUMZ_MP00168	Sangker River, Siem Reap, Cambodia	12°57’06.4”N, 103°08’45.4"E
	PRSEA160-20	BOLD:AEC9524	CUMZ_MP00177	Banteay Srei, Siem Reap, Cambodia	13°35’43.1”N, 103°57’42.6"E
	PRSEA161-20	BOLD:AEC9524	CUMZ_MP00178	Banteay Srei, Siem Reap, Cambodia	13°35’43.1”N, 103°57’42.6"E
*M*. *neglectum*	PRSEA056-20	BOLD:ADH8552	CUMZ_MP00073	Mueang, Krabi, Thailand	8°04’48.9”N, 98°55’08.5"E
	PRSEA057-20	BOLD:ADH8552	CUMZ_MP00074	Mueang, Satun, Thailand	6°44’43.5”N, 100°02’08.3"E
	PRSEA065-20	BOLD:ADH8552	CUMZ_MP00082	Khlong Yai, Trat, Thailand	11°54’15.1”N, 102°48’37.5"E
	PRSEA084-20	BOLD:ADH8552	CUMZ_MP00101	Mueang, Ranong, Thailand	9°53’13.6”N, 98°38’01.4"E
	PRSEA090-20	BOLD:ADH8552	CUMZ_MP00107	Mueang, Phangnga, Thailand	8°30’48.0”N, 98°30’13.2"E
*M*. *niphanae*	PRSEA017-20	BOLD:AAX3492	CUMZ_MP00034	Fak Tha, Uttaradit, Thailand	17°59’48.8”N, 100°52’44.0"E
	PRSEA019-20	BOLD:AAX3492	CUMZ_MP00036	Fak Tha, Uttaradit, Thailand	17°59’48.8”N, 100°52’44.0"E
	PRSEA022-20	BOLD:AAX3492	CUMZ_MP00039	Lom Sak, Phetchabun, Thailand	16°43’46.9”N, 101°14’17.0"E
	PRSEA023-20	BOLD:AAX3492	CUMZ_MP00040	Fak Tha, Uttaradit, Thailand	17°59’48.8”N, 100°52’44.0"E
	PRSEA038-20	BOLD:AAX3492	CUMZ_MP00055	Tha Yang, Phetchaburi, Thailand	12°56’55.8”N, 99°51’16.5"E
	PRSEA040-20	BOLD:AAX3492	CUMZ_MP00057	Thung Song, Nakhon Si Thammarat, Thailand	8°13’59.5”N, 99°40’32.1"E
	PRSEA069-20	BOLD:AAX3492	CUMZ_MP00086	Nam Pat, Uttaradit, Thailand	17°43’46.8”N, 100°41’24.3"E
	PRSEA109-20	BOLD:AAX3492	CUMZ_MP00126	Noen Maprang, Phitsanulok, Thailand	16°43’43.6”N, 100°35’10.3"E
	PRSEA146-20	BOLD:AAX3492	CUMZ_MP00163	Mueang, ‎Chai Nat, Thailand	15°13’20.6”N, 100°06’07.0"E
	PRSEA166-20	BOLD:AAX3492	CUMZ_MP00183	Banteay Srei, Siem Reap, Cambodia	13°35’43.1”N, 103°57’42.6"E
	PRSEA167-20	BOLD:AAX3492	CUMZ_MP00184	Banteay Srei, Siem Reap, Cambodia	13°35’43.1”N, 103°57’42.6"E
	PRSEA168-20	BOLD:AAX3492	CUMZ_MP00185	Banteay Srei, Siem Reap, Cambodia	13°35’43.1”N, 103°57’42.6"E
	GBCMD28534-19	BOLD:AAX3492	MF622022	Suphan Buri, Thailand*	
	GBCMD28535-19	BOLD:AAX3492	MF622023	Suphan Buri, Thailand*	
	GBCMD28536-19	BOLD:AAX3492	MF622024	Suphan Buri, Thailand*	
*M*. *nipponense*	PRSEA172-20	BOLD:AEB4023	CUMZ_MP00189	Dien Bien Phu, Vietnam	21°23’15.2”N, 103°00’46.8"E
	PRSEA173-20	BOLD:AEB4023	CUMZ_MP00190	Dien Bien Phu, Vietnam	21°23’15.2”N, 103°00’46.8"E
	PRSEA176-20	BOLD:AEB4023	CUMZ_MP00193	Dien Bien Phu, Vietnam	21°23’15.2”N, 103°00’46.8"E
	PRSEA177-20	BOLD:AEB4023	CUMZ_MP00194	Dien Bien Phu, Vietnam	21°23’15.2”N, 103°00’46.8"E
*M*. *palmopilosum*	PRSEA010-20	BOLD:AEC6469	CUMZ_MP00027	Bo Kluea, Nan, Thailand*	
	PRSEA012-20	BOLD:AEC6469	CUMZ_MP00029	Song Khwae, Nan, Thailand*	
	PRSEA020-20	BOLD:AEC8012	CUMZ_MP00037	Pong, Phayao, Thailand*	
	PRSEA021-20	BOLD:AEC8012	CUMZ_MP00038	Pong, Phayao, Thailand*	
	PRSEA027-20	BOLD:AEC8012	CUMZ_MP00044	Rong Kwang, Phrae, Thailand*	
	PRSEA029-20	BOLD:AEC8012	CUMZ_MP00046	Song, Phrae, Thailand*	
	PRSEA030-20	BOLD:AEC6469	CUMZ_MP00047	Chiang Klang, Nan, Thailand*	
*M*. *puberimanus*	PRSEA137-20	BOLD:ADX8426	CUMZ_MP00154	Na Yung, Udon Thani, Thailand*	
	PRSEA138-20	BOLD:ADX8426	CUMZ_MP00155	Pak Chom, Loei, Thailand*	
	PRSEA087-20	BOLD:ADX8426	CUMZ_MP00104	Chiang Khan, Loei, Thailand*	
	PRSEA106-20	BOLD:ADX8426	CUMZ_MP00123	Phu Ruea, Loei, Thailand*	
	GBCMD28531-19	BOLD:ADX2465	MF622019	Petchabun, Thailand*	
	GBCMD28530-19	BOLD:ADX8426	MF622018	Mukdahan, Thailand*	
*M*. *rogersi*	PRSEA097-20	BOLD:AEC8219	CUMZ_MP00114	La-Ngu, Satun, Thailand	6°54’22.3”N, 99°48’42.0"E
	PRSEA098-20	BOLD:AEC8219	CUMZ_MP00115	Mueang, Trang, Thailand	7°36’30.5”N, 99°33’47.8"E
	PRSEA107-20	BOLD:AEC8219	CUMZ_MP00124	La-Ngu, Satun, Thailand	6°54’22.3”N, 99°48’42.0"E
*M*. *rosenbergii*	GBCMD28532-19	BOLD:AAE0347		Suphan Buri, Thailand*	
	PRSEA083-20	BOLD:AAE0347	CUMZ_MP00100	Mueang, Maha Sarakham, Thailand	16°11’01.4”N, 103°27’24.4"E
	PRSEA100-20	BOLD:AAE0347	CUMZ_MP00117	Khlung, Chanthaburi, Thailand	12°28’00.0”N, 102°12’11.6"E
	PRSEA101-20	BOLD:AAE0347	CUMZ_MP00118	Mueang, Ranong, Thailand	9°53’13.5”N, 98°38’01.2"E
*M*. *sintangense*	PRSEA005-20		CUMZ_MP00022	Mueang, Phetchabun, Thailand	16°23’41.8”N, 101°10’15.5"E
	PRSEA018-20	BOLD:ADX3382	CUMZ_MP00035	Mueang, Phetchabun, Thailand	16°23’41.8”N, 101°10’15.5"E
	PRSEA033-20	BOLD:ADX3382	CUMZ_MP00050	Mueang, Prachin Buri, Thailand	16°23’41.8”N, 101°10’15.5"E
	PRSEA034-20	BOLD:ADX3382	CUMZ_MP00051	Kabin Buri, Prachin Buri, Thailand	13°56’14.7”N, 101°55’19.3"E
	PRSEA035-20	BOLD:ADX3382	CUMZ_MP00052	Khuan Don, Satun, Thailand	6°48’11.7”N, 100°05’28.7"E
	PRSEA036-20	BOLD:ADX3382	CUMZ_MP00053	Bo Rai, Trat, Thailand	12°23’48.4”N, 102°39’15.0"E
	PRSEA037-20	BOLD:ADX3382	CUMZ_MP00054	Bang Ban, Phra Nakhon Si Ayutthaya, Thailand	14°24’27.9”N, 100°28’13.0"E
	PRSEA041-20	BOLD:ADX3382	CUMZ_MP00058	Mueang, Nakhon Sawan, Thailand	15°42’11.6”N, 100°08’30.4"E
	PRSEA043-20	BOLD:ADX3382	CUMZ_MP00060	Mueang, Nakhon Nayok, Thailand	14°16’28.2”N, 101°16’59.0"E
	PRSEA062-20	BOLD:AAX3483	CUMZ_MP00079	Pak Chom, Loei, Thailand	18°01’04.2”N, 101°53’23.4"E
	PRSEA067-20	BOLD:ADX3382	CUMZ_MP00084	Kui Buri, Prachuap Khiri Khan, Thailand	12°05’29.0”N, 99°48’18.2"E
	PRSEA071-20	BOLD:ADX3382	CUMZ_MP00088	Sathing Phra, Songkhla, Thailand	7°30’36.3”N, 100°24’24.4"E
	PRSEA075-20	BOLD:ADX3382	CUMZ_MP00092	Sawi, Chumphon, Thailand	10°12’39.8”N, 99°03’55.5"E
	PRSEA081-20	BOLD:ADX3382	CUMZ_MP00098	Mueang, Trang, Thailand	7°36’30.5”N, 99°33’47.8"E
	PRSEA082-20	BOLD:ADX3382	CUMZ_MP00099	Mueang, Trang, Thailand	7°36’30.5”N, 99°33’47.8"E
	PRSEA092-20	BOLD:ADX3382	CUMZ_MP00109	Khlung, Chanthaburi, Thailand	12°27’59.9”N, 102°12’11.8"E
	PRSEA093-20	BOLD:ADX3382	CUMZ_MP00110	Suk Samran, Ranong, Thailand	9°23’28.3”N, 98°28’19.3"E
	PRSEA094-20	BOLD:AAX3483	CUMZ_MP00111	Chiang Khan, Loei, Thailand	17°54’15.7”N, 101°40’51.2"E
	PRSEA095-20	BOLD:ADX3382	CUMZ_MP00112	Soi Dao, Chanthaburi, Thailand	13°07’12.6”N, 102°13’02.5"E
	PRSEA105-20	BOLD:ADX3382	CUMZ_MP00122	Mueang, Chanthaburi, Thailand	12°35’11.4”N, 102°06’05.0"E
	PRSEA155-20	BOLD:ADX3382	CUMZ_MP00172	Tonle Sap River, Kandal, Cambodia	11°49’01.8”N, 104°48’35.3"E
	PRSEA185-20	BOLD:AAX3483	CUMZ_MP00202	Khemarat, Ubon Ratchathani, Thailand	16°02’37.1”N, 105°13’28.3"E
	PRSEA188-20	BOLD:ADX3382	CUMZ_MP00205	Takeo, Cambodia	10°58’51.2”N, 104°46’43.2"E
	PRSEA190-20	BOLD:ADX3382	CUMZ_MP00207	Prek Thraot River, Kampong Speu, Cambodia	11°27’33.0”N, 104°31’31.1"E
	PRSEA186-20	BOLD:AAX3479	CUMZ_MP00203	Dan Chang, Suphan Buri, Thailand	14°50’57.0”N, 99°40’09.0"E
	PRSEA187-20	BOLD:ADX3382	CUMZ_MP00204	Tonle Sap Lake, Cambodia	12°30’22.1”N, 104°27’17.2"E
	PRSEA193-20	BOLD:AAX3483	CUMZ_MP00210	Muang Khong, Champasak, Laos	14°06’55.4”N, 105°51’20.0"E
	PRSEA194-20	BOLD:AAX3483	CUMZ_MP00211	Muang Khong, Champasak, Laos	14°06’55.4”N, 105°51’20.0"E
	PRSEA195-20	BOLD:AAX3483	CUMZ_MP00212	Pakse, Champasak, Laos	5°07’56.0”N, 105°48’39.5"E
	PRSEA196-20	BOLD:AAX3483	CUMZ_MP00213	Muang Khong, Champasak, Laos	14°06’55.4”N, 105°51’20.0"E
	GBCMD2448-09	BOLD:AAX3483	FM958083	Warin Chamrap, Thailand*	
	GBCMD28539-19	BOLD:ADX3382	MF622026	Suphan Buri, Thailand*	
	GBCMD28538-19	BOLD:ADX3382	MF622025	Suphan Buri, Thailand*	
	GBCMD28537-19	BOLD:ADX3382	MF622027	Suphan Buri, Thailand*	
*M*. *sirindhorn*	PRSEA007-20	BOLD:AEC8029	CUMZ_MP00024	Chiang Kham, Phayao, Thailand*	
	PRSEA008-20	BOLD:AEC8029	CUMZ_MP00025	Chiang Kham, Phayao, Thailand*	
	PRSEA009-20	BOLD:AEC8029	CUMZ_MP00026	Chiang Kham, Phayao, Thailand*	
	PRSEA110-20	BOLD:AEC8029	CUMZ_MP00127	Chiang Kham, Phayao, Thailand*	
*M*. *spelaeus*	PRSEA142-20	BOLD:AEC6618	CUMZ_MP00159	Noen Maprang, Phitsanulok, Thailand	16°40’51.2"N 100°41’27.4"E
	PRSEA143-20	BOLD:AEC6618	CUMZ_MP00160	Noen Maprang, Phitsanulok, Thailand	16°40’51.2"N 100°41’27.4"E
*M*. *suphanense*	GBCMD28543-19	BOLD:ADX7811	MF622031	Kanchanaburi, Thailand*	
	GBCMD28542-19	BOLD:ADX7811	MF622030	Suphan Buri, Thailand*	
	GBCMD28541-19	BOLD:ADX7813	MF622029	Suphan Buri, Thailand*	
	GBCMD28540-19	BOLD:ADX7812	MF622028	Suphan Buri, Thailand*	
	PRSEA184-20	BOLD:ADX7812	CUMZ_MP00201	Nong Chang, Uthaitani, Thailand	15°23’17.9”N, 99°51’14.5"E
*M*. *thai*	PRSEA059-20	BOLD:AEC7291	CUMZ_MP00076	Mueang, ‎Chaiyaphum, Thailand	15°57’19.4”N, 102°02’01.1"E
	PRSEA060-20	BOLD:AEC7291	CUMZ_MP00077	Wang Sam Mo, Udon Thani, Thailand	16°56’49.7”N, 103°28’11.8"E
	PRSEA061-20	BOLD:AEC7291	CUMZ_MP00078	Phon Phisai, Nong Khai, Thailand	17°59’41.4”N, 103°03’48.8"E
*M*. *tratense*	PRSEA032-20	BOLD:ADX3382	CUMZ_MP00049	Bo Rai, Trat, Thailand	12°23’48.4”N, 102°39’15.0"E
	PRSEA104-20	BOLD:ADX3382	CUMZ_MP00121	Bo Rai, Trat, Thailand	12°23’48.4”N, 102°39’15.0"E
*M*. *trompii*	GBCMD2447-09	BOLD:AAX3494	FM958084	Riau, Indonesia*	
*M*. *villosimanus*	PRSEA099-20	BOLD:AEC9637	CUMZ_MP00116	La-ngu, Satun, Thailand	6°54’22.3”N, 99°48’42.2"E
*M*. *yui*	PRSEA068-20	BOLD:AEC7099	CUMZ_MP00085	Mueang, Mae Hong Son, Thailand	19°25’09.9"N 97°59’53.2"E
	PRSEA102-20	BOLD:AEC7099	CUMZ_MP00119	Mueang, Mae Hong Son, Thailand	19°25’09.9"N 97°59’53.2"E
	PRSEA103-20	BOLD:AEC7099	CUMZ_MP00120	Mueang, Mae Hong Son, Thailand	19°25’09.9"N 97°59’53.2"E
Putative sp. 1	GBCM16528-19	BOLD:ADW8174	MH053369	Chainat, Thailand, Thailand*	
Putative sp. 2	GBCMD28533-19	BOLD:ADW5335	MF622021	Suphan Buri, Thailand*	
Putative sp. 3	PRSEA079-20	BOLD:AEC6743	CUMZ_MP00096	Mueang, Maha Sarakam, Thailand	16°11’01.4”N, 103°27’24.4"E
	PRSEA080-20	BOLD:AEC6743	CUMZ_MP00097	Udonthani, Thailand	17°19’02.5”N, 102°35’53.0"E
Putative sp. 4	PRSEA003-20	BOLD:AED0644	CUMZ_MP00020	Nam Pat, Uttaradit, Thailand	17°43’47.0”N, 100°41’24.3"E
	PRSEA006-20	BOLD:AED0644	CUMZ_MP00023	San Sai, Chiang Mai, Thailand	18°53’59.1”N, 99°00’41.1"E
	PRSEA126-20	BOLD:AED0644	CUMZ_MP00143	Mueang, Krabi, Thailand	8°04’49.1”N, 98°55’08.6"E
	PRSEA127-20	BOLD:AED0644	CUMZ_MP00144	Bo Rai, Trat, Thailand	12°23’48.4”N, 102°39’15.0"E
	PRSEA128-20	BOLD:AED0644	CUMZ_MP00145	Suan Phueng, Ratchaburi, Thailand	13°32’55.9”N, 99°17’25.8"E
	PRSEA129-20	BOLD:AED0644	CUMZ_MP00146	Klaeng, Rayong, Thailand	12°47’05.7”N, 101°40’59.6"E
putative sp. 5	PRSEA174-20	BOLD:AEC9626	CUMZ_MP00191	Dien Bien Phu, Vietnam	21°23’15.2”N, 103°00’46.5"E
putative sp. 6	GBCMD2470-09	BOLD:AAX6071	FM958061	Chiangmai, Thailand*	
Putative sp. 7	PRSEA189-20	BOLD:AAK6470	CUMZ_MP00206	Preaet Tuek Chhu River, Kampot, Cambodia	10°33’43.6”N, 104°04’41.4"E
Putative sp. 8	GBCMD2457-09	BOLD:AAX3487	FM958074	Nee Soon, Singapore*	
Putative sp. 9	GBCMD2451-09	BOLD:AAX3479	FM958080	Tonle Sap, Cambodia*	
	PRSEA180-20	BOLD:AAX3479	CUMZ_MP00197	Bang Ban, Phra Nakhon Si Ayutthaya, Thailand	14°24’27.9”N, 100°28’13.0"E
	PRSEA191-20	BOLD:AAX3479	CUMZ_MP00208	Chhnok Tru, Kampong Chhnang, Cambodia	12°30’21.1”N, 104°27’17.0"E
	PRSEA192-20	BOLD:AAX3479	CUMZ_MP00209	Chongchom, Kap Choeng, Surin, Thailand	14°27’11.2”N, 103°41’29.2"E
Putative sp. 10	PRSEA096-20	BOLD:AEC6997	CUMZ_MP00113	Khong Chiam, Ubon Ratchathani, Thailand	15°19’13.3”N, 105°29’51.7"E
Putative sp. 11	PRSEA178-20	BOLD:AEC6999	CUMZ_MP00195	Tha Tum, Surin, Thailand	15°19’32.0”N, 103°40’28.6"E
	PRSEA179-20	BOLD:AED0856	CUMZ_MP00196	Tha Uthen, Nakhon Phanom, Thailand	17°34’31.2”N, 104°36’12.4"E
	PRSEA181-20	BOLD:AED0856	CUMZ_MP00198	Mueang, ‎Mukdahan, Thailand	16°32’27.7”N, 104°43’04.1"E
Putative sp. 12	PRSEA182-20		CUMZ_MP00199	Mueang, ‎Maha Sarakam, Thailand	16°11’01.4”N, 103°27’24.4"E
	PRSEA183-20	BOLD:AEC6998	CUMZ_MP00200	Mueang, ‎Maha Sarakam, Thailand	16°11’01.4”N, 103°27’24.4"E
Putative sp. 13	PRSEA169-20	BOLD:AED0058	CUMZ_MP00186	Paksong, Champasak, Laos	15°11’49.7”N, 106°06’24.1"E
Putative sp. 14	PRSEA170-20	BOLD:AEB5988	CUMZ_MP00187	Paksong, Champasak, Laos	15°11’49.7”N, 106°06’24.1"E
	PRSEA171-20	BOLD:AEB5988	CUMZ_MP00188	Paksong, Champasak, Laos	15°11’49.7”N, 106°06’24.1"E
	PRSEA039-20	BOLD:AEB5988	CUMZ_MP00056	Bueng Khong Long, Bueng Kan, Thailand	17°58’11.8”N, 104°02’24.3"E
	GBCM16418-19	BOLD:AEB5988	MH053368	Beung Kan, Thailand*	
Putative sp. 15	PRSEA158-20	BOLD:AED0766	CUMZ_MP00175	Phumi Phsar River, Kampong Chhnang, Cambodia	12°22’51.5”N, 104°28’59.6"E
	PRSEA159-20	BOLD:AED0766	CUMZ_MP00176	Phumi Phsar River, Kampong Chhnang, Cambodia	12°22’51.5”N, 104°28’59.6"E
	PRSEA163-20	BOLD:AED0766	CUMZ_MP00180	Phumi Phsar River, Kampong Chhnang, Cambodia	12°22’51.5”N, 104°28’59.6"E
	PRSEA164-20	BOLD:AED0766	CUMZ_MP00181	Stung Sen River, Kampong Thom, Cambodia	12°42’34.7”N, 104°52’24.6"E
	PRSEA165-20	BOLD:AED0766	CUMZ_MP00182	Chinit River, Kampong Thom, Cambodia	12°47’27.7”N, 104°49’13.4"E
Putative sp. 16	PRSEA115-20	BOLD:AEC7050	CUMZ_MP00132	Wang Wiset, Trang, Thailand	7°38’43.8”N, 99°31’55.4"E
Putative sp. 17	PRSEA028-20	BOLD:AEC6619	CUMZ_MP00045	Pong, Phayao, Thailand	19°07’11.1”N, 100°16’49.8"E
Putative sp. 18	PRSEA047-20	BOLD:AEC9332	CUMZ_MP00064	Phanom, Surat Thani, Thailand*	15° 13’ 19.8192"N, 100° 6’ 6.1158"E
	PRSEA064-20	BOLD:AEC9332	CUMZ_MP00081	Rattaphum, Songkhla, Thailand*	15° 13’ 19.8192"N, 100° 6’ 6.1158"E
	PRSEA085-20	BOLD:AED0022	CUMZ_MP00102	Xishuangbanna, China*	16° 39’ 2.88"N, 101° 46’ 22.8354"E
	PRSEA086-20	BOLD:AED0022	CUMZ_MP00103	Xishuangbanna, China*	16° 49’ 20.6364"N, 100° 25’ 56.5392"E
	PRSEA120-20	BOLD:AED0409	CUMZ_MP00137	Si Sawat, Kanchanaburi, Thailand*	19°07’11.1”N, 100°16’49.8"E
	PRSEA121-20	BOLD:AEC9331	CUMZ_MP00138	Wang Thong, Phitsanulok, Thailand*	13°48’33.6”N, 102°03’16.8"E

The names of OTUs retrieved from the consensus delimitation methods.

*record in literature: Saengphan, Panijpan [70] Siriwut, Jeratthitikul [88] and Wowor, Muthu [100].

### Species delimitation

Species delimitation was performed using four standardized methods: automated barcode gap (ABGD; Puillandre, Lambert [102]), the multi-rate Poisson Tree Processes (mPTP, Kapli, Lutteropp [103]), Bayesian implementation of Poisson Tree Processes model (bPTP, Zhang, Kapli [104]), and the Generalized Mixed Yule Coalescent model (GMYC, Fujisawa and Barraclough [105]). For the ABGD method, the initial distance of intra- and interspecific variations obtained from each sequence was calculated in MEGA 7 and the optimized barcode relative gap analysis was implemented using the ABGD online sever (http://wwwabi.snv.jussieu.fr/public/abgd/abgdweb.html). All parameters were configured as default settings except X (relative gap width), which was set as 1. The operating distance used in ABGD was calculated with Kimura (K80) TS/TV method. The graphical ratio between the number of OTUs and prior interspecific divergence was established to obtain the optimized threshold for barcode gap designation. The equivalent phase of recursive and initial partitions was selected to depict the number of putative OTUs found in the delimitation result.

The mPTP analysis was conducted using the online server (http://mptp.h-its.org). The ML tree generated from RAxML in Nerwick format was uploaded to the web server. The outgroup selection was initially designated for the purpose of tree rooting. The visualization of the final delimitation tree was set as default. The bPTP species delimitation was carried out by online sever (https://species.h-its.org/). The Bayesian tree was initially calculated in MrBayes under 10 million sampling generations. The tree result was then transformed to either NEXUS or Nerwick format and submitted to the web server. All parameters such as number of MCMC generation and burn-in were set as default. The OTU clustering was estimated under 95% confidence of statistical probability.

In the GMYC method, the initial Bayesian tree was calculated in BEAST v1.10.4 package [106, 107] using a Mixed-Yule coalescent model tree prior. The sequence dataset was transformed into NEXUS format. All parameter settings were configured in BEAUTi v1.8.4. Tracer v1.6 was used to check ESS values and run the trace file. The construction of an ultra-metric tree was done in BEAST v1.10.4 via the CIPRES sever [95]. The maximum clade supported tree result was summarized in TreeAnnotator v1.10.4. The GMYC species delimitation was performed using R program with “splits” package [108].

### Integrative taxonomic decision scheme

The results of delimitation methods can be varied due to the criteria of justification: ABGD requires the threshold value from genetic distance, GMYC is processed under ultramatric estimation of gene tress, PTP uses the same fundamental estimation as GMYC does but the effect of branch length proportion and the amount of genetic change is implied. Each molecular method result may be affected by population size, number of species involved, species divergence and number of sampling singletons in dataset. For morphological delimitation, the effect of geographical variation and limitation of samples for comparative study interfered with the species discrimination that reached variable OTU number in delimitation results. For this reason, we implemented the consensus criteria of OTU counts to summarize the final result. The results of five species delimitation methods were compared to find the consensus results, including **1)** the traditional morphological identification, **2)** molecular phylogenetic clade support from ML and BI reconstruction methods based on COI gene datasets (≥ 70 bootstrap value for ML and ≥ 0.95 posterior probability for BI), **3)** the distance gap of COI barcode analysis in BOLD and ABGD, **4)** the Poisson Tree Processes model under multi-rate and Bayesian implementation (mPTP and bPTP), and **5)** the generalized mixed Yule-coalescent (GMYC). The delimitation criteria for putative clustering were established based on a 66% consensus of five methods [109]. The species boundary was discriminated when the delimitation results showed congruence in at least four of the five methods. Species boundary lines were illustrated on a delimitation tree. Exemption criteria were implied in cases of singleton OTUs of representative taxa in datasets of this study. The BIN discordance result was established to serve as a warning signal for further morphological re-examination and taxonomic revision.

## Results

### Morphological identification and species occurrences

**Fig 2:**
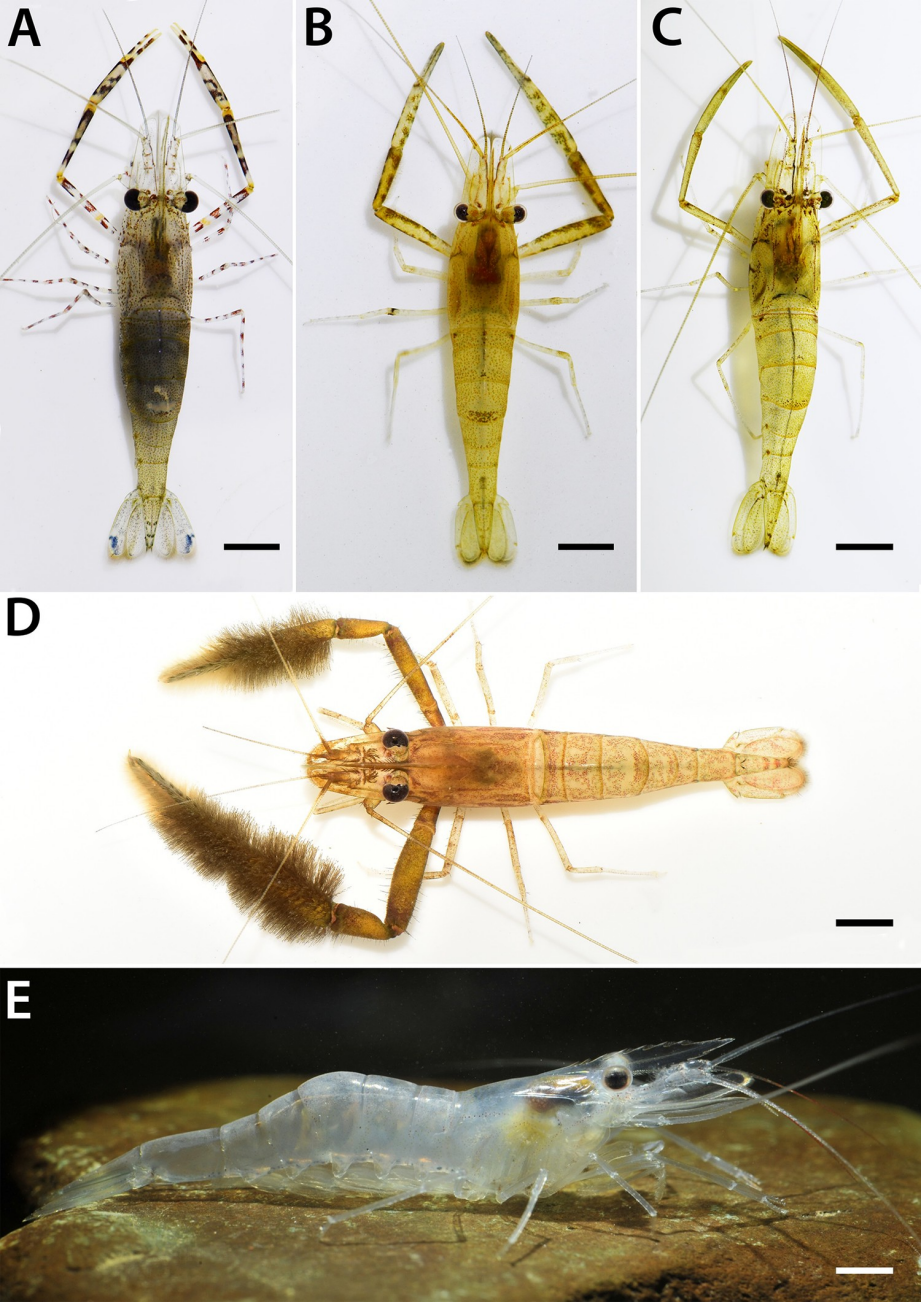
Representative morphology and live habitus coloration of five common *Macrobrachium* species; A. *M*. *equidens* B. *M*. *niphanae* C. *M*. *sintangense* D. *M*. *dienbienphuense* and E. *M*. *lanchesteri*. Approximate size shown in scale bar (5 mm).

Twenty-four nominal species from newly collected materials were successfully identified based on comparative morphology using various taxonomic sources. In total, thirty-six morphological species were included in the dataset of this study including identified specimens with deposited sequences from previous molecular taxonomic work. Pictures of some common morphological species groups found in the Indochina subregion are presented in Fig 2. The diagnostic characters have been evaluated and additional taxonomic characters have been recorded. The main taxonomic characters used in species identification were rostrum shape, rostrum teeth, epistome, anteromedial spine on carapace, second pereiopods, uropodal diaresis, and shape of telson. However, some taxonomic characters were highly variable in some geographical populations. In this study, juvenile specimens of some *Macrobrachium* species such as *M*. *chainatense*, *M*. *lanchesteri*, *M*. *niphanae*, *M*. *nipponense*, *M*. *sintangense*, and *M*. *suphanense* presented similarities in their morphological features, including rostrum shape, rostrum teeth number, and the modification pattern of second pereiopods. This illustrates the problem of taxonomic ambiguity in specimen identification and species richness determination in localities where only juvenile specimens were collected. Moreover, taxonomic ambiguity was also detected for adult specimens in several common and widespread species such as *M*. *dienbienphuense*, *M*. *eriocheirum* [currently synonymized as *M*. *dienbienphuense*; in Li, Liu [110]], *M*. *forcipatum*, *M*. *lanchesteri*, *M*. *malayanum*, *M*. *niphanae*, *M*. *nipponense*, *M*. *sintangense*, and *M*. *suphanense*. The diagnostic characters for some species showed a high proportion of overlap with other species living in the same area, such as number of teeth on fingers of second perieopods, the structure and number of rostrum teeth, and the presence of velvet pubescence on second perieopods. Nevertheless, the integration of morphology and molecular phylogenetic analysis (see below) represented an effective means to clarify the species boundaries among these prawn species.

### Molecular phylogeny

**Fig 3:**
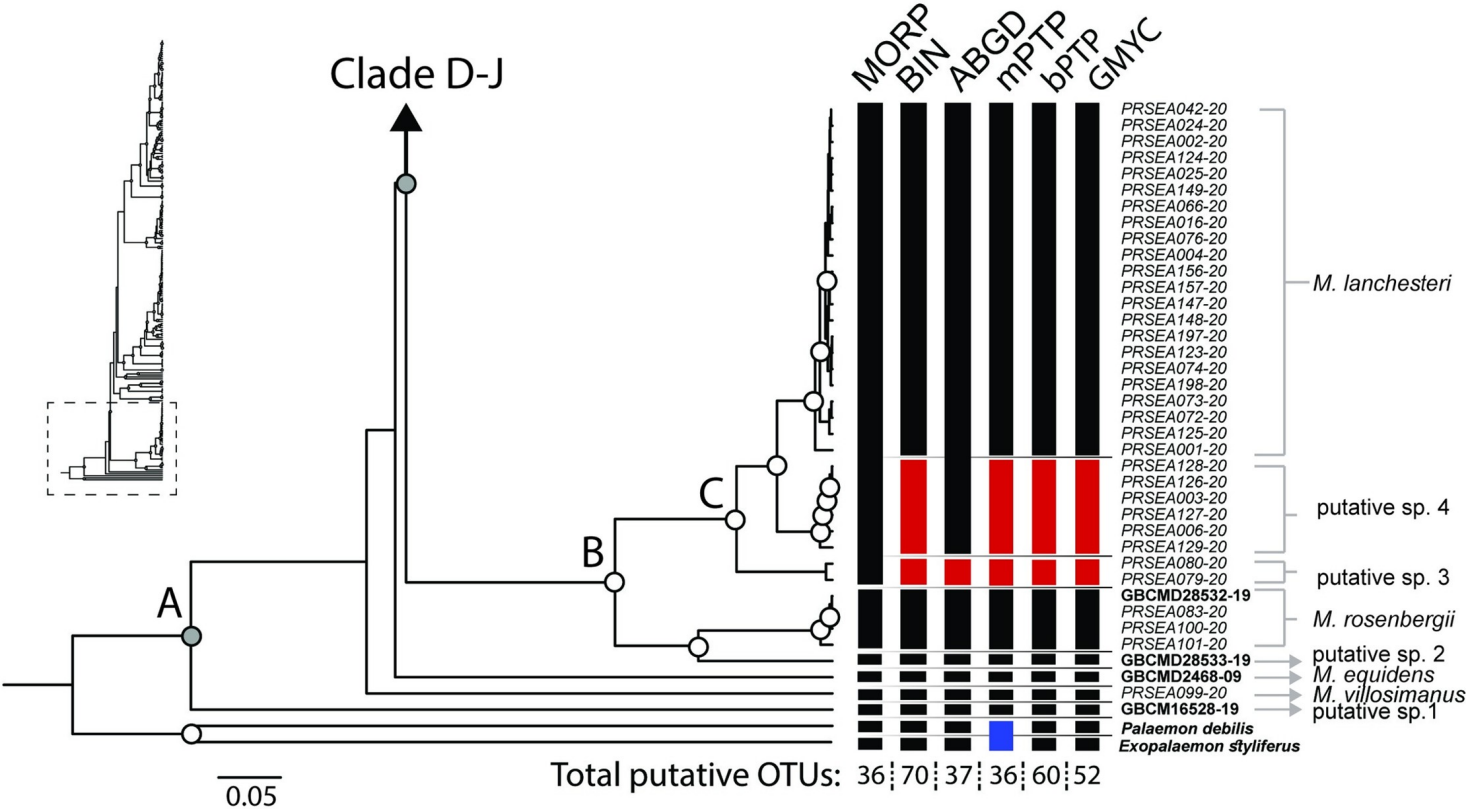
Ultrametric tree of Indochinese *Macrobrachium* indicating clade support level and species delimitation clustering results (part 1). Abbreviations used on tree are as follows: Morpho, morphological identification; BIN, BIN method in BOLD; ABGD, automated barcode gap; bPTP, Bayesian Poisson tree processes; mPTP, multi-rate Poisson tree processes; GMYC, generalized mixed Yule coalescent model. Box colors indicate split (red) and lumped (blue) species recognized by each species delimitation method. Grey boxes indicate missing sequences. Morphological data from dataset in each delimitation method; sample ID with bold style indicates samples obtained from BOLD and NCBI. The horizontal line and species labels show the consensus result of species clustering.

**Fig 4:**
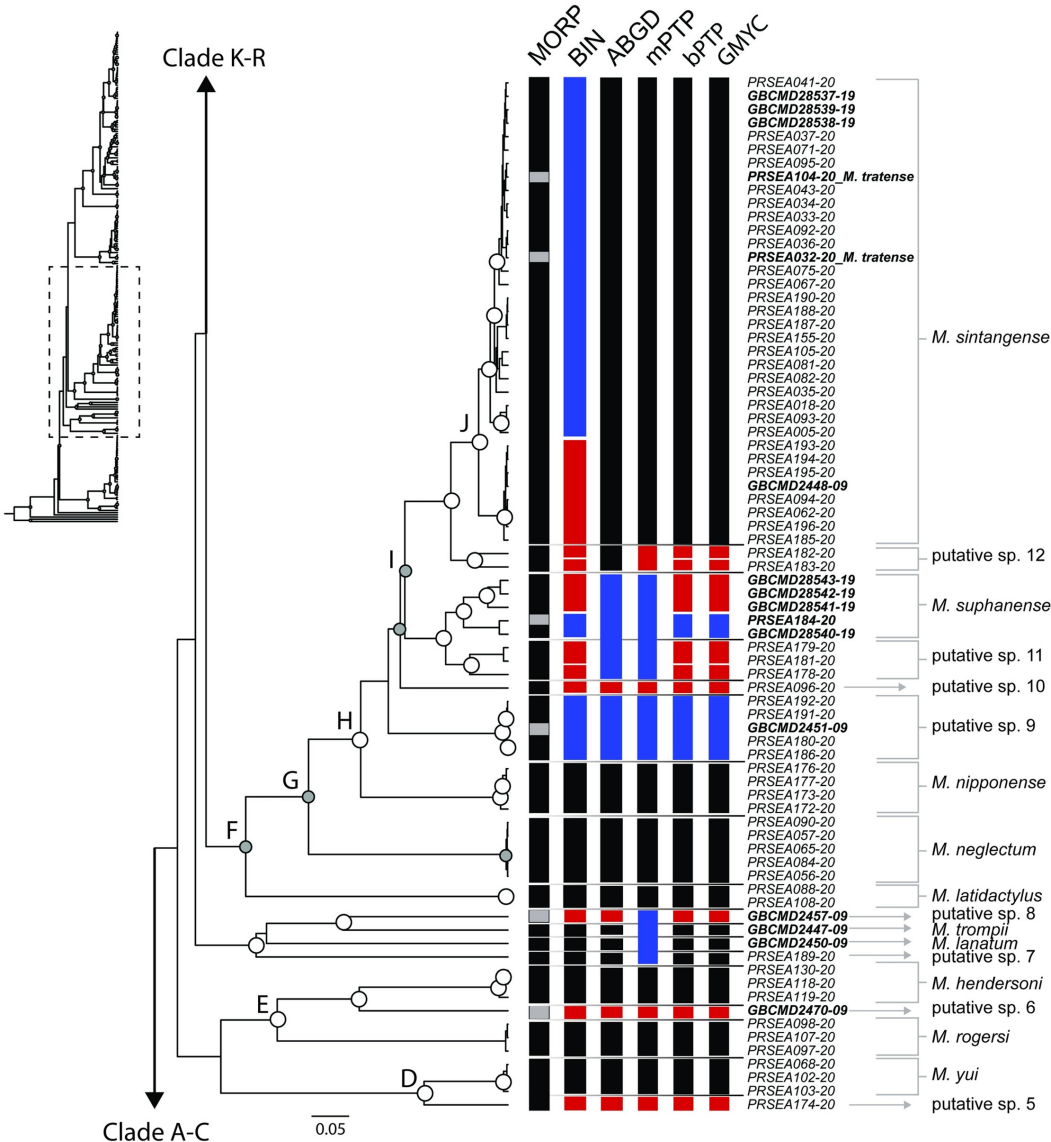
Ultrametric tree of Indochinese *Macrobrachium* indicating clade support level and species delimitation clustering results (part 2). For abbreviations used on tree see Fig 3.

**Fig 5:**
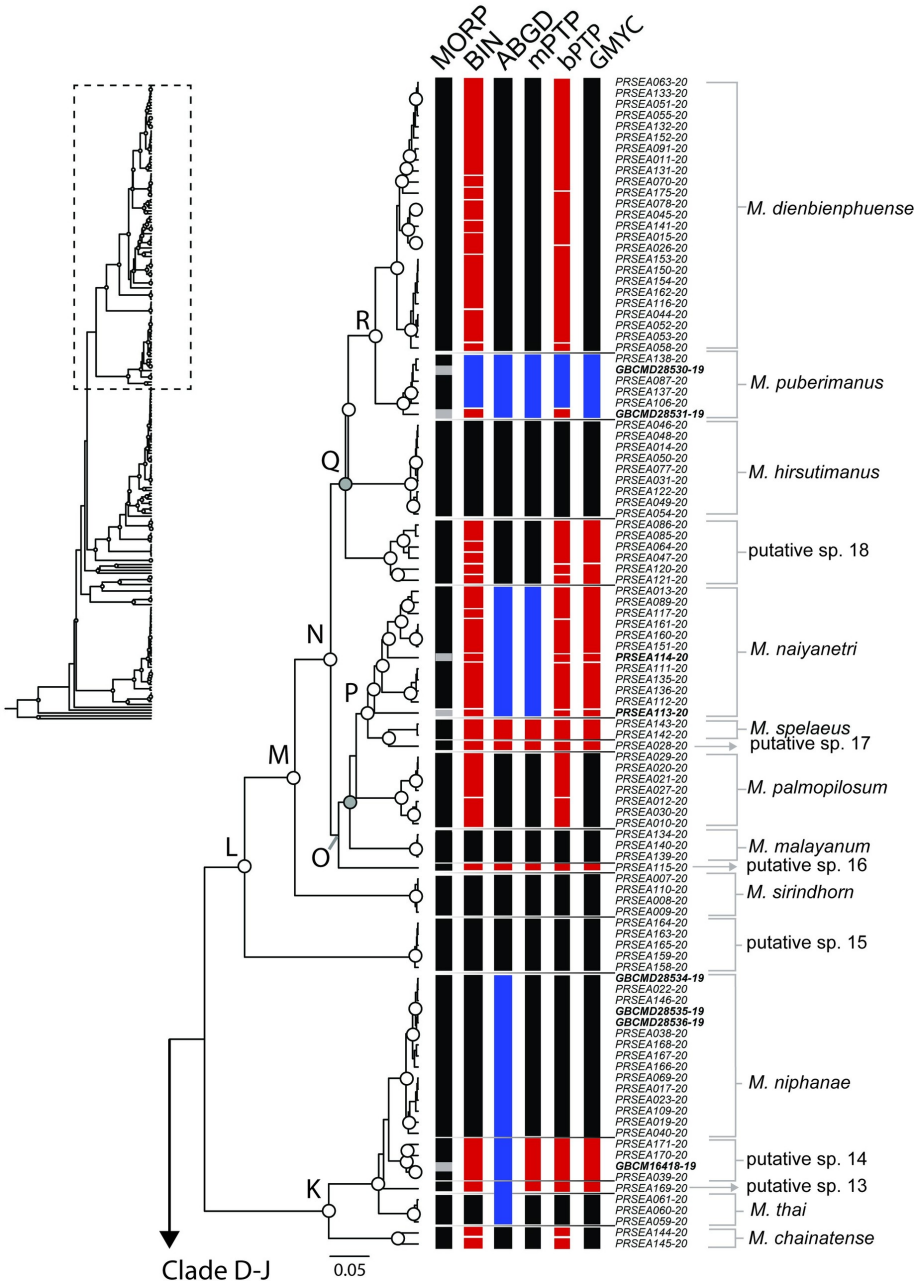
Ultrametric tree of Indochinese *Macrobrachium* indicating clade support level and species delimitation clustering results (part 3). For abbreviations used on tree see Fig 3.

One hundred ninety-eight partial sequences of COI gene were successfully obtained. The final aligned dataset contained a total of 678 bp, which included 326 conservative sites, 352 variable sites, and 284 parsimony-informative sites. All sequences were deposited in the BOLD database. BOLD identification numbers are presented in Table 2. The phylogenetic tree and clade composition compiled from three reconstruction methods was illustrated on an ultrametric topology (Figs 3–5). According to the accepted criteria for a monophyletic clade, the bootstrap value in RAxML failed to surpass the threshold value, whereas W-IQ-TREE and BI tree both supported monophyly. However, phylogenetic relationships among some *Macrobrachium* species are unresolved due to low statistical support at the deep node. The topology supports monophyly in most *Macrobrachium* species sampled in this study. The ultrametric tree depicts monophyletic clade clustering of samples of eighteen nominal species: *M*. *chainatense*, *M*. *hendersoni*, *M*. *hirsutimanus*, *M*. *lanchesteri*, *M*. *latidactylus*, *M*. *naiyanetri*, *M*. *neglectum*, *M*. *niphanae*, *M*. *nipponense*, *M*. *palmopilosum*, *M*. *puberimanus*, *M*. *rosenbergii*, *M*. *rogersi*, *M*. *sintangense*, *M*. *suphanense*, *M*. *sirindhorn*, *M*. *spelaeus*, *M*. *thai*, and six putative species: putative sp. 4, 9, 12, 14 to15, and 18. Specimens identified as *M*. *dienbienphuense*, *M*. *lanchesteri*, *M*. *niphanae*, *M*. *sintangense*, and *M*. *yui* are shown to be highly genetically diversified, and populations are split into several lineages. Some newly amplified sequences of specimens morphologically identical to *M*. *sintangense* are represented as a polyphyletic group due to the insertion of the *M*. *suphanense* sequence within the clade of *M*. *sintangense*.

Within the *Macrobrachium* clade (Fig 3: clade A), two previously deposited and one newly amplified sequence (GBCM16528-19, GBCMD2468-09, and PRSEA099-20), represented as putative sp. 1, *M*. *equidens*, and *M*. *villosimanus*, are placed in a basal position with low statistical support. *Macrobrachium rosenbergii*, *M*. *lanchesteri* and three putative lineages are grouped into clade B. *Macrobrachium lanchesteri* exhibits a clade structural pattern with three deeply divergent lineages (clade C). Moreover, one deposited *M*. *lanchesteri* sequence (GBCMD28533-19) is assembled within a clade of *M*. *rosenbergii*.

Three *Macrobrachium* species, *M*. *hendersoni*, *M*. *rogersi*, and *M*. *yui* are closely clustered (Fig 4), but the relationship has low statistical support from both ML and BI. Inland freshwater and estuarine species are clustered in clades F-J, including *M*. *neglectum*, *M*. *latidactylus*, *M nipponense*, *M*. *sintangense*, and *M*. *suphanense*. The major clade of *M*. *sintangense* (clade J) is shown to have shallow genetic divergence and is differentiated from two specimens from Northeast Thailand, defined as putative sp. 12 (PRSEA183-20 and PRSEA182-20). A relationship between *M*. *sintangense*-putative sp. 12 and *M*. *suphanense* is identified. Moreover, the incongruence of two nominal species, *M*. *saigonense* (GBCMD2451-09) and *M*. *tratense* (PRSEA104-20 and PRSEA032-20) is detected due to the insertion of these sequences within *M*. *sintangense* lineages.

Fig 5 shows that clade K represents the monophyletic clusters of *M*. *niphanae*, *M*. *thai*, and *M*. *chainatense*. Shallow genetic structure of *M*. *niphanae* is detected. The delimitation methods suggest two additional putative groups placed closely to *M*. *niphanae* lineages (putative sp. 13 and 14). Clade L harbors a group of *Macrobrachium* with high morphological diversification. Twelve distinct lineages of *Macrobrachium* species including seven putative species form a monophyletic relationship. Putative sp. 15 is placed as a basal lineage, followed by *M*. *sirindhorn* specimens (clade M). The rest of *Macrobrachium* is separated into two major lineages (node O and clade Q). Phylogenetic position of *M*. *malayanum* is not monophyletic due to one distinct sequence with low clade composition creditability support, which is instead assigned by delimitation method to be a new putative singleton sample (putative sp. 16).

*Macrobrachium spelaeus* and *M*. *naiyanetri* show a close relationship with statistical support from both BI and ML analyses (clade P). The samples previously defined as *M*. *forcipatum* (based on morphology; PRSEA114-20, PRSEA113-20) are inserted within *M*. *naiyanetri*. In clade Q, the ultrametric tree unites the four monophyletic clades among species including putative sp. 18, *M*. *dienbienphuense*, *M*. *hirsutimanus*, and *M*. *puberimanus*. In clade R, a widespread species in Indochina, namely *M*. *dienbienphuense*, is united with *M*. *puberimanus*. However, the two previously deposited samples of *M*. *dienbienphuense* (GBCMD28530-19 and GBCMD28531-19) are placed inside the *M*. *puberimanus* cluster.

In this study, some deposited sequences of SE-Asian *Macrobrachium* species reported in previous studies are combined in the ultrametric tree, including *M*. *equidens*, *M*. *lanatum*, *M*. *malayanum*, *M*. *rosenbergii*, *M*. *trompii*, and *M*. *yui*. Surprisingly, some sequences are shown as isolated lineages, and not joined congruently with new material based on taxonomic identification in this study, i.e., *M*. *dienbienphuense* (GBCMD28530-19 and GBCMD28531-19), *M*. *forcipatum* (PRSEA113-20 and PRSEA114-20), *M*. *lanchesteri* (GBCMD28532-19), *M*. *malayanum* (GBCMD2457-09), and *M*. *yui* (GBCMD2470-09). Furthermore, the monophyletic relationship of representative OTUs of *M*. *rogersi* is rejected, probably caused by long-branch attraction of singleton species or similar haplotype sampling conditions.

### DNA barcoding

All new sequences deposited in BOLD and GenBank database are shown in Table 2. The project code for barcode analysis was accounted as “PRSEA” in BOLD. All COI sequences successfully passed the check for possible insertion of stop codons. The nucleotide composition of COI fragments used in analysis is as follows. The composition frequency of nucleotides is G: 18.91±0.07%, C: 25.94±0.22%, A: 26.71±0.14%, and T: 28.44±0.14%. The GC content of each codon position is 54.59 ±0.15% in position 1, 44.20±0.012% in position 2, 35.74±0.73% in position 3 and 44.84±0.29% overall. The genetic divergence distances calculated from 222 sequences retrieved from 36 morphological species in the dataset are 5.52% and 20.25% within species and genus, respectively. The normalized within-species divergence is 3.06±0.12%.

**Fig 6:**
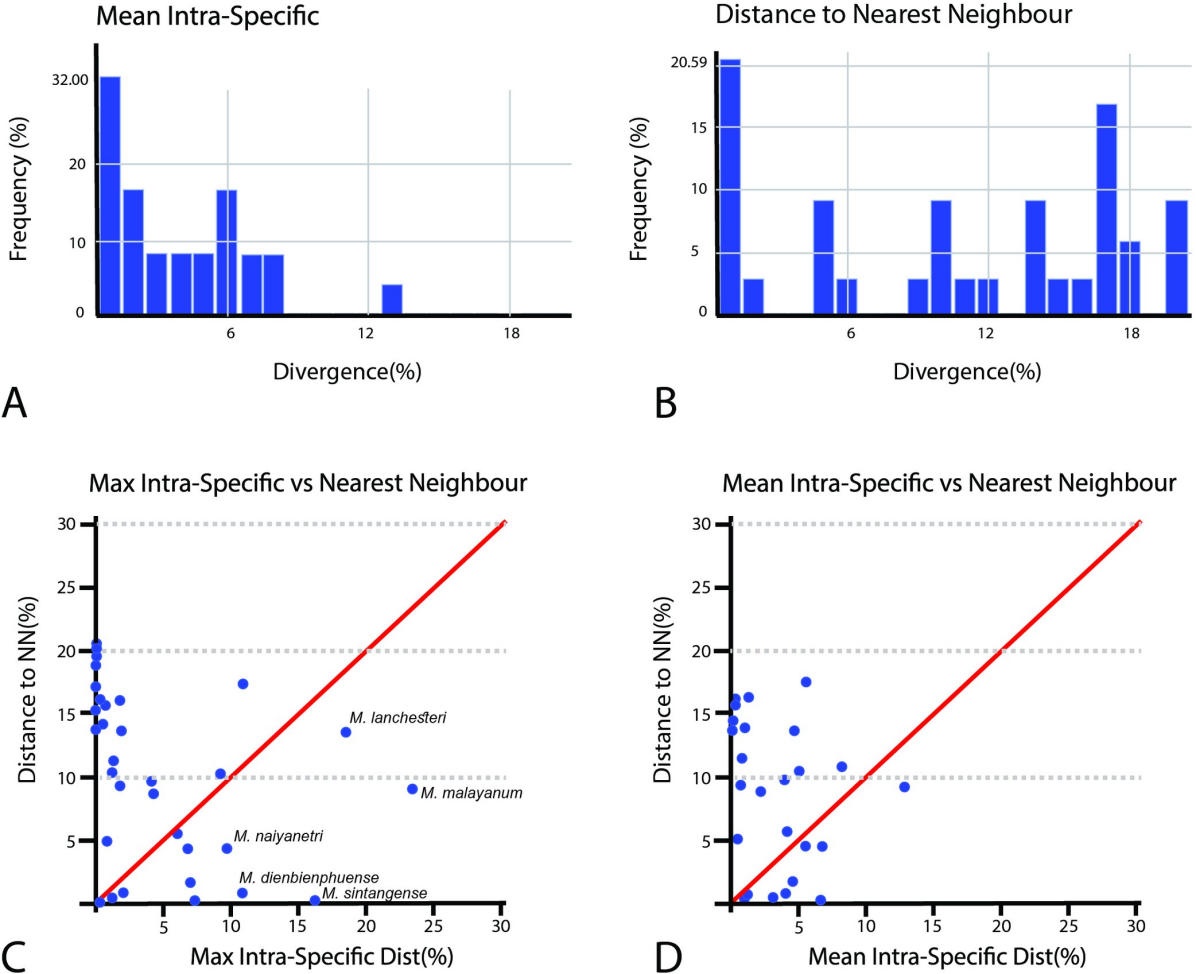
Results of genetic distance obtained from BOLD under K2-parameter model; A. Mean intra sequence divergence B. Distance to nearest-neighbor species based on sequence divergence C. Comparison of maximum intraspecific variation and distance to nearest-neighbor species D. Comparison of mean intraspecific variation and distance to nearest-neighbor species.

The barcode gap analysis based on comparison between mean/max-intraspecific variation and nearest-neighbor distance was calculated using two alternative models: K2-P and P-distance. The comparison results for singleton species were excluded due to non-applicable data in calculations. In K2-P and P-distance models, the species comparison advocated fourteen species with both max-intra and mean-intra distances higher than nearest neighboring species. A scatter plot warning of high genetic divergence species based on K2-P distances is provided in Fig 6 and a summary of barcode gap comparisons among all *Macrobrachium* species is presented in Table 3.

**Table 3:** Summary of Mean-Max intraspecific variation in each *Macrobrachium* species and the distance to nearest-neighbor species.

NO.	Species	Mean Intra-Sp.	Max Intra-Sp.	Nearest Species	Nearest Neighbor	Distance to NN
1	*M*. *chainatense*	3.97	3.97	*M*. *niphanae*	PRSEA038-20	9.85
2	*M*. *dienbienphuense*	3.94	10.94	*M*. *puberimanus*	PRSEA138-20	0.6
3	*M*. *equidens*	N/A	0	*M*. *sintangense*	PRSEA096-20	20.06
4	*M*. *forcipatum*	6.69	6.69	*M*. *naiyanetri*	PRSEA117-20	4.44
5	*M*. *hendersoni*	1.31	1.61	putative sp. 5	GBCMD2470-09	16.55
6	*M*. *hendersoni*	5	9.06	*M*. *hirsutimanus*	PRSEA122-20	10.56
7	*M*. *hirsutimanus*	0.79	1.8	*M*. *spelaeus*	PRSEA028-20	9.53
8	*M*. *lanatum*	N/A	0	*M*. *sintangense*	PRSEA186-20	15.77
9	*M*. *lanchesteri*	4.79	18.54	*M*. *rosenbergii*	PRSEA101-20	14.02
10	*M*. *latidactylus*	0.3	0.3	*M*. *sintangense*	PRSEA192-20	16.56
11	*M*. *malayanum*	12.87	23.49	*M*. *spelaeus*	PRSEA028-20	9.23
12	*M*. *naiyanetri*	5.59	9.61	*M*. *forcipatum*	PRSEA114-20	4.44
13	*M*. *neglectum*	0	0	*M*. *nipponense*	PRSEA177-20	13.95
14	*M*. *niphanae*	2.95	7.25	putative sp. 14	GBCM16418-19	0.15
15	*M*. *nipponense*	0.84	1.34	*M*. *sintangense*	PRSEA096-20	11.52
16	*M*. *palmopilosum*	2.22	4.12	*M*. *spelaeus*	PRSEA028-20	8.85
17	*M*. *puberimanus*	1.17	1.8	*M*. *dienbienphuense*	GBCMD28530-19	0.6
18	*M*. *rogersi*	0.2	0.3	*M*. *suphanense*	GBCMD28543-19	16.52
19	*M*. *rosenbergii*	1	1.83	*M*. *lanchesteri*	GBCMD28533-19	14.02
20	*M*. *saigonense*	N/A	0	*M*. *sintangense*	PRSEA192-20	0
21	*M*. *sintangense*	6.62	16.02	*M*. *saigonense*	GBCMD2451-09	0
22	*M*. *sirindhorn*	0.22	0.44	*M*. *naiyanetri*	PRSEA135-20	14.69
23	*M*. *spelaeus*	4.05	6.08	*M*. *forcipatum*	PRSEA114-20	5.6
24	*M*. *suphanense*	4.62	7.02	*M*. *sintangense*	PRSEA184-20	1.52
25	*M*. *suphanense*	N/A	0	*M*. *sintangense*	PRSEA184-20	20.9
26	*M*. *thai*	0.49	0.74	*M*. *niphanae*	PRSEA171-20	4.92
27	*M*. *tratense*	1.04	1.04	*M*. *sintangense*	PRSEA095-20	0.15
28	*M*. *trompii*	N/A	0	*M*. *naiyanetri*	PRSEA112-20	17.76
29	*M*. *villosimanus*	N/A	0	*M*. *suphanense*	GBCMD28540-19	19.36
30	*M*. *yui*	5.64	10.96	*M*. *hirsutimanus*	PRSEA077-20	17.85
31	putative sp. 5	N/A	0	*M*. *hendersoni*	PRSEA118-20	16.55
32	putative sp. 7	N/A	0	*M*. *neglectum*	PRSEA090-20	16.67
33	putative sp. 14	N/A	0	*M*. *niphanae*	PRSEA039-20	0.15
34	putative sp. 15	0.24	0.59	*M*. *spelaeus*	PRSEA142-20	16.05

Grey highlighting indicates taxa with warning signal of genetic divergence retrieved from barcode gap analysis.

The DNA diagnostic characters in *Macrobrachium* species with a minimum of three representative sequences in the input dataset were successfully detected. The five categories of nucleotide characters were default assigned: diagnosis, partial or diagnosis, partial or uninformative, invalid and uninformative characters. The frequency of each character level found in the dataset are as follows: six species contained diagnosis character, one species with diagnosis or partial character, and 15 species with partial character. Partial-uninformative and invalid characters were not detected in any of the COI sequences in this study. The COI sequences of *M*. *sirindhorn* and *M*. *rogersi* contained four and five diagnosis characters, respectively. The nucleotide position and assigned character category of selected *Macrobrachium* species are summarized in S1 Table.

The BIN discordance analysis indicated five cluster groups which contained non-confirmation of sequence cluster validity based on the BIN comparison of input records within the cluster. These BIN clusters contained previously deposited sequences and the newly obtained sequence dataset from this study. The discordant BIN clusters were identified as follows: *M*. *dienbienphuense–M*. *puberimanus* (BOLD:ADX8426), *M*. *sintangense–M*. *suphanense* (BOLD:ADX7812), *M*. *niphanae–Macrobrachium* sp.1SS-2018 (BOLD:AEB5988), *M*. *sintangense–M*. *tratense* (BOLD:ADX3382) and *M*. *sintangense–M*. *saigonense* (BOLD:AAX3479).

### Species delimitation

**Fig 7:**
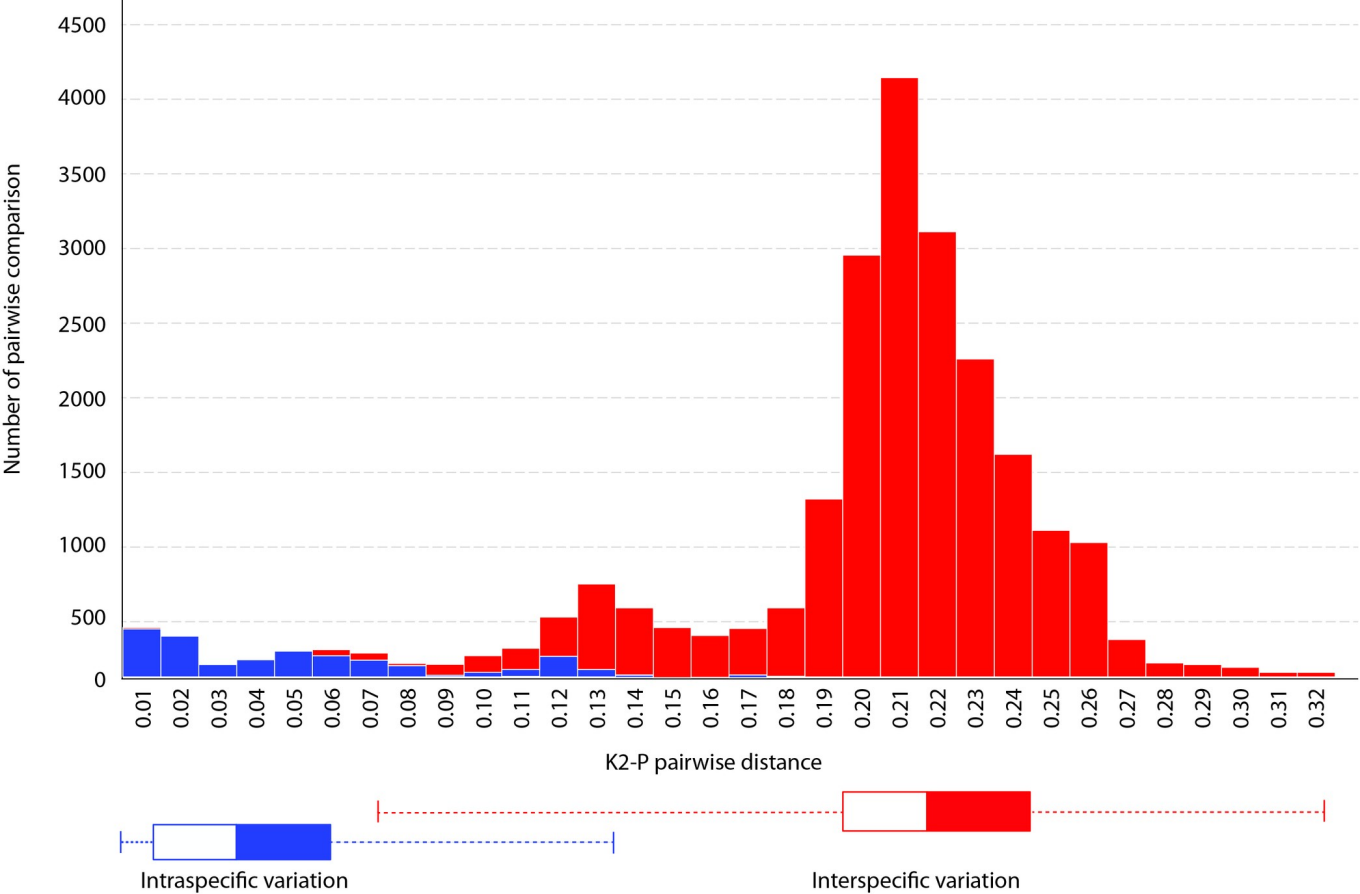
Results of barcoding gap representing intra- and inter-specific variations of *Macrobrachium* species retrieved from BOLD and this study.

The ABGD method delimited the sequence dataset into 37 MOTU clusters (excluding outgroup taxa). The clustering assigned nine putative lineages from singleton samples. The barcoding gaps calculated from pairwise comparisons of COI sequences are shown in Fig 7. The number of putative MOTUs, counted in the BIN clustering method, are represented by a total of 70 clusters. The BIN algorithm intensively suggested deep genetic divergence in several species such as *M*. *sintangense*, *M*. *lanchesteri*, *M*. *dienbienphuense* and putative sp. 6 to 10 and 14. The Poisson tree process (mPTP) indicated 36 MOTUs, which was lower than the result from bPTP delimitation. The bPTP depicted 31 additional MOTUs on the tree. The highest MOTU count was detected in clade M, likely the result of BIN delimitation. The GMYC method marked 21 additional MOTUs and showed similarlity with BIN and bPTP results by high intensity designation of *Macrobrachium* species in clade M.

Furthermore, five delimitation methods revealed the combination of previously deposited sequences from different species. In all methods, a clade comprised of *M*. *tratense* and *M*. *sintangense* was designated as single cluster (Fig 4: clade J). The BIN algorithm marked three lumped locations (blue box in Figs 4 and 5) belonging to samples of five species, namely *M*. *dienbienphuense*, *M*. *saigonense*, *M*. *sintangense*, *M*. *suphanense*, and *M*. *tratense*. In ABGD, five lumped locations were detected under composite clades of *M*. *saigonense-M*. *sintangense*, *M*. *sintangense*-*M*. *suphanense*, *M*. *niphanae-M*. *thai*, *M*. *forcipatum*-*M*. *naiyanetri*, *M*. *dienbienphuense*-*M*. *puberimanus*. In mPTP, the detection of clustering yield showed the highest number of recomposed clades. Nine designated groups showed contradictory results from traditional morphological identification due to multiple insertions of samples in different species or genera. The bPTP and GMYC methods depicted a similar lumped pattern among paired species as follows: in *M*. *sintangense*-*M*. *saigonense*, *M*. *sintangense*-*M*. *suphanense*, *M*. *dienbienphuense*-*M*. *puberimanus*.

In total, the five delimitation methods, including morphological and molecular schemes, assigned the number of putative OTUs from the input COI dataset (including outgroups) as follows: 36 groups by morphology, 70 groups by BIN, 37 groups by ABGD, 36 groups by mPTP, 60 groups by bPTP, and 52 groups by GMYC. The delimitation results from each method are illustrated in Figs 3–5 based on topology of the ultrametric tree. The consensus result based on an integrative taxonomic scheme designated 27 nominal and 18 putative species from the COI dataset (excluding two outgroups).

## Discussion

### Cryptic diversity and morphological variation of *Macrobrachium* prawns in Indochina

In this study, *Macrobrachium* COI sequences retrieved twenty-seven nominal and eighteen unknown putative species under the consensus tree generated from traditional morphology and molecular phylogenetic affinities. Specimens referred to *M*. *dienbienphuense*, *M*. *forcipatum*, *M*. *lanchesteri*, *M*. *malayanum*, *M*. *saigonense*, *M*. *sintangense*, *M*. *tratensae*, and *M*. *yui* show some discordance. Morphological examination indicated problematic diagnostic characters used among those species by showing them to be highly variable. For example, **1)** the number of rostrum teeth has a broad range of variation in some long-distance dispersal species such as *M*. *dienbienphuense*, *M*. *lanchesteri*, and *M*. *niphanae*; **2)** features of the second pereiopod such as spinulation on cuticular surfaces of telopodites, velvet and pubescence, number of teeth on chela and pollex show variation among populations and species; **3)** length and shape variation in the second pereiopod podomeres such as the carpus and merus seem to vary among geographical populations and are related to sexual dimorphism (e.g., *M*. *dienbienphuense* and *M*. *sirindhorn*). The transitional zones of variation between and within species showed a high degree of overlap (Fig 7). Possible cryptic diversification based on high intraspecific divergence was detected in several species such as *M*. *lanchesteri* (23.68%), *M*. *malayanum* (23.49%), and *M*. *sintangense* (16.34%). The barcode gap analysis in BOLD depicts 13 clustering species having maximum intraspecific distance greater than the distance to their nearest-neighbor species in the dataset (Table 3). Cryptic speciation seems to occur within species groups with morphological complexity and co-existing distributions.

From delimitation results, unknown putative OTUs with morphological variation and high genetic divergence were detected in several nominal species such as *M*. *dienbienphuense*, *M*. *forcipatum*, *M*. *lanchesteri*, *M*. *niphanae*, *M*. *sintangense*, *M*. *spelaeus*, and *M*. *suphanense*. The tree topology also indicates a genetic structural pattern correlated to geographical distinction in several widespread species. *Macrobrachium lanchesteri*, a common and widespread species, is represented by two morphological patterns based on the following composite characters: the proportional length of palm and finger of second pereiopods, rostrum shape, and body size. In the ultrametric tree, the *M*. *lanchesteri* clade is shown as two geographically distinct putative groups (putative sp. 3 and 4, Fig 3), which are supported by both BI and ML analyses. The geographical differentiation of *M*. *lanchesteri* has been previously reported, based on karyotypes [111], microsatellite markers, traditional morphometry, and single locus phylogeny [112]. In the case of rostrum form, *M*. *lanchesteri* closely resembles other species such as *M*. *idea*, *M*. *peguense* and *M*. *tiwarii*. Because body length and size in *M*. *lanchesteri* show such variability, specimens in adult and juvenile stages are likely confused with morphologically similar species living in the same area, such as *M*. *niphanae*, *M*. *nipponense*, *M*. *rosenbergii*, *M*. *sintangense*, and *M*. *thai*.

The long-distance dispersal species, *M*. *sintangense*, and the recently described species, *M*. *suphanense* [70], are widely distributed in the Chao Phraya Basin, and some populations may migrate into the Mekong Basin. The phylogenetic results reveal several deeply divergent lineages, and delimitation methods cluster four new putative groups and place them between *M*. *sintangense* and *M*. *suphanense* (Fig 4). Another conspecific species closely resembling *M*. *sintangense* and *M*. *suphanense* is *M*. *nipponense*, which has been reported as an introduced species in northern Laos, Vietnam, and Myanmar [49, 50, 53, 113]. In this study, sequences of *M*. *nipponense* from Vietnam were successfully sequenced and validated with a previous taxonomic study of Vietnamese fauna [53]. It is unclear whether the distribution of *M*. *nipponense* in Indochina is due to human introduction or native migration. However, a recent study on phylogeography and population structure of *M*. *nipponense* between coastal China and the island of Taiwan provides evidence that migration was associated with the Pleistocene glacial cycle and ecological isolation within a lake [60].

In Indochina, there are four additional *Macrobrachium* species that likely belong in this group: *M*. *dolatum* and *M*. *tratense* described from Thailand and two Mekong River species, *M*. *saigonense* and *M*. *hungi*, described from the lower Mekong Basin, in the Tonle Sap Basin in Cambodia and the Mekong Delta in southern Vietnam. These four species morphologically resemble *M*. *sintangense*. Only one deposited sequence of *M*. *saigonense* was available (GBCMD2451-09) to be included in this study. The delimitation result suggested the insertion of *M*. *saigonense* within the distinct lineage of putative sp. 9, which was previously identified as *M*. *sintangense* based on morphology. This result indicates the need for taxonomic reassessment of these two morphologically similar species. The life history traits of these species show association with estuarine environments and their morphological characters of the second pereiopods show similarly pattern (by having long and slender telopodites with 1–3 large teeth on chela and pollex). However, the inclusive phylogenetic relationship is unresolved due to low support values in BI and ML. Further revision of taxonomic descriptions and distribution patterns of these species is required.

The two remaining species groups found in this study, i.e., *M*. *niphanae* sensu Hanamura, Imai [50] and *M*. *pilimanus* sensu Johnson [114] exhibit high genetic and morphological divergence. The ultrametric tree advocates three species belonging to the *M*. *niphanae* group (Fig 5: clade K): *M*. *chainatense*, *M*. *niphanae*, and *M*. *thai*, whereas the clade of *M*. *pilimanus* (clade M) contains 12 clustered species. According to phylogenetic positions among members within clade K, the position of *M*. *chainatense* is rooted as the basal lineage from *M*. *niphanae*, *M*. *thai*, and two putative species. *Macrobrachium niphanae* and *M*. *thai* share similar morphological characters, but they were reported separately and with different geographical distributions: *M*. *niphanae* was reported from central Thailand and Laos, whereas *M*. *thai* was narrow distributed in the Korat plateau, Northeast Thailand [48, 115]. Delimitation methods agree with the distinction of *M*. *niphanae* from *M*. *thai*, except the AGBD method. Moreover, BIN, ABGD, mPTP, bPTP, and GMYC delimit two distinct lineages within *M*. *niphanae* (putative sp. 13 and 14). Previously, *M*. *meini*, with morphological characters resembling *M*. *niphanae*, was reported to be distributed in central and northeastern Thailand, and in Laos. This species probably coexists with *M*. *niphanae* and *M*. *thai* in several habitats in Indochina [48].

For the clade composed of the *M*. *pilimanus* group (clade L), genetic divergence shows congruence with delimitation approaches by having OTU counts that seem higher than from traditional identification. Recently, the *M*. *pilimanus* group was taxonomically examined for its morphological variability [50]. Arguments regarding species validity were discussed due to geographical variability, and the group was re-investigated because of suspected misidentification of name-bearing types [48, 50, 116, 117]. In this study, two patterns of geographical distributions are detected in mainland SE-Asian species, namely the Chao Phraya and Mekong Basins (Fig 5: clade N), although statistical support is lacking for the monophyly of the Chao Phraya group (Fig 5: node O). In the Mekong Basin group (clade Q), tree topology indicates two nominal and two putative species.

*Macrobrachium dienbienphuense* is the most widespread species of the *M*. *pilimanus* group in Indochina and is placed in the Mekong Basin group. The taxonomic problem of morphological variability was reported in *M*. *dienbienphuense*, and insufficient species delimitation was attributed to the effect of geographical variation [48, 50, 110]. In this study, species delimitation approaches indicate one putative OTU, putative sp. 18. The Chao Phraya group also contains two new distinct lineages (putative sp. 16 and 17). Unexpectedly, the new distinct lineage from Cambodian territory (putative sp. 15) appears as a basal clade of the *M*. *pilimanus* group. This result suggests the need for further taxonomic revision and multi-locus phylogenetic study in order to clarify the taxon validity and to explore cryptic diversity of the *M*. *pilimanus* species group in Indochina.

*Macrobrachium yui* and *M*. *hendersoni*, both land-locked species, are distributed in the northwestern montane area of Indochina with occurrence reported from two major river basins: Chao Phraya and Mekong [48, 50]. Distribution ranges of these species and other nominal taxa such as *M*. *lanatum* and *M*. *patheinense* [49, 118] serve as evidence of exchange between Indochinese and Indian-Burmese fauna due to populations found in the Indian subcontinent. In this study, seven species with cross-basin distribution were found: *M*. *hendersoni*, *M*. *lanchesteri*, *M*. *neglectum*, *M*. *nipponense*, *M*. *rogersi*, *M*. *rosenbergii*, and *M*. *vilosimanus*. The transitional zone of these freshwater species exists along the montane and coastal area of Myanmar-Thailand border. This result advocates that the diversity of Indochinese fauna might be underestimated due to habitat diversification and the connection of river networks. For this reason, the biogeographical study between Indochina and other regions such as India-Burma, East Asia, and some Sunda Islands of the Malay Archipelago should emphasize evolutionary history at a broad regional scale. Moreover, the taxonomic revision of several species previously reported from Laos, Cambodia and Vietnam requires reinvestigation using an integrative taxonomic approach.

### Implications of DNA barcode delimitation of groups with high morphological diversification in genus *Macrobrachium*

Barcode-based delimitation provides an additional tool for cryptic diversity exploration and can be used to resolve taxonomic validity in morphologically complex organisms including crustacean decapods [35, 44, 119–123]. Previously, the proposal of a universal threshold for species delimitation in crustaceans using genetic divergence has been introduced [39]. In decapods, the barcode delimitation approach has also been used in particularly important groups such as crayfish [124], shrimp and prawns in families Atyidae and Palaemonidae [121, 125, 126]. The universal barcode gap threshold based on COI and 16s markers has been excavated by broad sampling analysis of crustacean families. The result suggested that the barcode gap threshold from the COI marker is helpful for taxonomy in species-level identification [39]. In this study, the barcode gap distance generated from MEGA provided the broad picture of overall distance values. The graph (Fig 7) can be used to explain the overlap between intra- and inter- specific distances either the best compromised barcode gap threshold value present clearly or hardly determine. A graph from BOLD provides the evidence of high-low genetic differentiation among sequence clusters. The BOLD graph is useful for further taxonomic reinvestigation because the fine resolution of clustering group determination and warning signal provided from BIN discordance. For this reason, barcode gap graphs might give a broad resolution of delimitation based on distance method in both best and compromised barcode gap threshold proportion. The median universal genetic divergence between species of the same genus is between 0.25–1.01 substitutions per site. In this study, the maximum interspecific divergence is 20.9, whereas maximum intraspecific divergence was as high as 23.49 (in *M*. *malayanum;* Table 2). This finding suggests the possibility of cryptic divergence in *Macrobrachium* species.

Morphological adaptations driven by intrinsic and extrinsic factors in shrimp and prawns have been reported worldwide [56, 127–129]. An impact on developmental stages in species with hermaphroditism was found when the food resource and space were limited [46]. Morphological variation such as phenotypic plasticity or sequential hermaphroditism in aquatic animals was reported in association with food supply and habitat space [58, 130]. These aforementioned factors also cause significant impact on species discrimination due to the degree of morphological overlap in *Macrobrachium* species [131, 132]. In this study, juvenile specimens of the *M*. *pilimanus* species group are difficult to identify because of the lack or incomplete development of species diagnostic characters generally used in broad sampling. Sexual dimorphism was documented in some *Macrobrachium* species, such as in a male with the pair of second perieopods showing different shape and length of each telopodite, whereas the female exhibited similar form on both sides. A lack of life history data is problematic for species identification as well as for potential use in aquaculture [133–135].

In this study, the BIN discordance function in BOLD provided a warning signal for taxa with low genetic distance compared to their nearest-neighbor species. For example, deposited COI sequences of *M*. *lanchesteri* and *M*. *dienbienphuense* exhibit genetic affinity and are possibly closely related to *M*. *rosenbergii* and *M*. *puberimanus* sequences (in this study), respectively. The taxonomic identification of *M*. *puberimanus* and *M*. *dienbienphuense* was clearly resolved based on multi-locus phylogenetic analysis despite their sympatric distribution patterns and morphological variability [88]. The morphological characters and molecular delimitation agree that these co-existing species must be accepted as distinct species. Moreover, the BIN discordance signal suggests that some *M*. *dienbienphuense* sequences (GBCMD28530-19 and GBCMD28531-19) placed within the *M*. *puberimanus* clade require re-investigation of their morphological characters for identification. Within clade B (Fig 3), one deposited sequence defined as *M*. *lanchesteri* (GBCMD 28533–19) is not placed within *M*. *lanchesteri* s.l. clade but rather as a sister lineage of *M*. *rosenbergii*.

Recent taxonomic revision has validated the taxonomic identity of *M*. *rosenbergii* [136]. In Indochina, the introduced population of *M*. *rosenbergii* stock from different geographical populations used in breeding programs may lead to genetic contamination among native populations. This result raises a signal of caution for specimen identification and use of Indochinese *Macrobrachium* sequences deposited in the online database. For this reason, the intensive study of riverine species is critically needed to clarify taxonomic boundaries and to document genetic diversity of native populations. In this study, barcode gap analysis also reveals the concealing effect of this method for species delimitation among Indochinese species due to high inter- and intra-specific genetic variations.

Recently, primers for the COI region were re-configured due to species-specification, improvement of amplification ability, and avoidance of nuclear copied gene amplification. However, the use of DNA barcoding has been debated for intensive systematic study [137–139]. The nuclear mitochondrial DNA (*numts*), a common DNA segment found in crustaceans, was suspected to interfere with the COI sequences used in crustacean phylogeny and barcode analysis [122, 140, 141]. The low quality of COI sequences containing *numts* may cause misleading results in species genealogies and clustering methods [142]. In this study, all COI sequences were automatically checked before BIN assignment in BOLD. The clustering result based on COI sequences was partially congruent with morphological species identification. The phylogenetic position of several morphological species such as *M*. *dienbienphuense*, *M*. *eriochierum*, *M*. *hirsutimanus*, *M*. *neglectum*, *M*. *niphanae*, *M*. *sirindhorn*, and *M*. *thai* were illustrated. However, the single-locus phylogenetic analysis was unable to resolve relationships in deeply divergent lineages of some nominal species. The integration of other molecular loci for decapod identification such as 16S, 28S, and H3 would allow improvement of taxonomic identification and study of phylogenetic relationships [15, 100]. According to DNA barcoding results, it can be suggested that taxonomic revision and assessment of phylogenetic relationships of mainland SE-Asian *Macrobrachium* including the Indochina subregion are crucially needed, especially in groups that show morphological complexity such as *M*. *lanchesteri*, *M*. *niphanae*, and *M*. *sintangense*. Moreover, deposited sequences in the public barcode library might be of significant importance for fundamental knowledge, utilization and future conservation management projects of mainland SE-Asian *Macrobrachium* fauna.

### Distribution patterns of Indochinese freshwater *Macrobrachium* prawns

The biogeography and life history of *Macrobrachium* in SE-Asia have been revealed based on the combination of molecular, morphological and ecological data [37, 38, 100]. The marine-freshwater habitat diversification has been suggested to have had an impact on morphological and genetic diversifications in aquatic animals [143–145]. Previously, life-history traits of *Macrobrachium* prawn species were matched with their evolutionary relationships by using multi-loci phylogenetic analysis [100]. The marine species with larval development in saline water were depicted as the ancestral group for all *Macrobrachium*, while the freshwater group appear to be the derivative group. In this study, the ultrametric tree depicts both marine-brackish and freshwater species. However, the fine resolution of phylogenetic relationships between species with the two life history traits remains undetermined due to low structural signal in the deep node position. Broad-scale phylogeographical studies of giant river prawn species and some Indo-Australian species [43, 146] suggested that the vast genetic diversity could be divided into several groups based on morphological complexity, and that the genetic affinity of some populations supported the hypothesis of an ancient river system during the last glacial maximum period [147, 148]. The historical connection of several rivers in mainland SE-Asia has been frequently detected in historical biogeographical studies in other aquatic organisms such as semi-aquatic earthworms [149], freshwater mussels [150, 151], fishes [152, 153], as well as from evidence in the fossil record, e.g., crocodilian [154].

From multiple lines of ecological field evidence in this study, together with habitat preferences and distribution ranges gleaned from previous records, the life history of Indochinese *Macrobrachium* are divided into three groups. The first group includes species found in montane streams and comprises seventeen taxa including nominal and putative clustering species: *M*. *dienbienphuense*, *M*. *forcipatum*, *M*. *hendersoni*, *M*. *hirsutimanus*, *M*. *malayanum*, *M*. *naiyanetri*, *M*. *palmopilosum*, *M*. *puberimanus*, *M*. *spelaeus*, *M*. *sirindhorn*, *M*. *yui* and putative sp. 5, 6, and 15 to 18. The second group includes estuarine species found among mangrove forests in the Andaman Sea and in the Gulf of Thailand: *M*. *equidens*, *M*. *villosimanus*, *M*. *neglectum* and putative sp. 7 to 11. The third group of species is distributed within inland tributaries of two major drainages of the Mekong and Chao Phraya rivers. This group comprises ten nominal and seven putative species: *M*. *chainatense*, *M*. *lanchesteri*, *M*. *latidactylus*, *M*. *niphanae*, *M*. *rogersi*, *M*. *rosenbergii*, *M*. *sintangense*, *M*. *suphanense*, *M*. *thai*, and putative sp. 1 to 4 and 12 to 14. Further analysis on habitat characteristics such as substrate type and water quality could be investigated to confirm these three life history traits in Indochina fauna.

Distribution patterns of some *Macrobrachium* groups show correlation with historical evidence supporting a paleo river systems hypothesis [37, 150]. In this study, the occurrence records of three *Macrobrachium* species could be used to refer historical geography. Firstly, *M*. *naiyanetri* exhibited disjunct distribution between the southern Thai peninsula, east coast of Thailand and inland water system of Cambodia. The genetic affinity based on clade composition of sampling population from those areas also supported the geographical isolation. Secondly, the subpopulation of *M*. *dienbienphuense* has been found along the western part of Thailand, whereas the major population seems distributed in Mekong tributaries. The previous hypothesis about the connection between minor tributary systems of Mekong River and Chaophraya River was discussed and promoted based on phylogeographic analyses of extant and extinct organisms (mentioned below). The records of occurrence for this species would support the river connection hypothesis. M. lanchesteri is commonly abundant in lentic water habitats in Indochina. In this study, the genetic affinities were detected and divided into three geographical areas. The genetic structure likely fits with three major river systems in Thailand (Chauphraya, Tapi and Mekong), which were previously part of the ancient river system during Pleistocene. Further investigation on population genetics and molecular dating would be useful to match species diversification with the effects of historical distribution scenarios.

From phylogenetic analysis, two widespread species, *M*. *neglectum* and *M*. *sintangense* exhibit low genetic divergence among different geographical populations. In contrast, the phylogenetic tree indicates geographical isolation among the populations of two widespread species, *M*. *dienbienphuense* and *M*. *lanchesteri*. Populations of *M*. *dienbienphuense* and *M*. *lanchesteri* often are found co-existing with other congeneric species. *Macrobrachium lanchesteri* are highly abundant in flood-plain areas, whereas *M*. *dienbienphuense* predominantly migrates within the Mekong River and its tributaries. Recently, the migratory period for *M*. *dienbienphuense* has been locally reported as “parading” in some remote areas in tributaries of the middle Mekong, and the species currently might be threatened by human activity [155]. The discovery of subterranean species such as *M*. *elegantum*, *M*. *lingyunense*, and *M*. *spelaeus* [63, 87] highlights the high adaptive ability to extreme habitats and emphasizes the significance of habitat diversity in continental Asia. Previous taxonomic studies and the results of DNA barcoding in this study advocate that the freshwater fauna of Indochina is still underestimated for cryptically diverse species because of river tributary connections [48–50, 156]. For this reason, the distribution patterns of several *Macrobrachium* species in Indochina are evidence that help solve the puzzle of SE-Asian freshwater faunal diversity related to a paleo-river basin presented in previous reports. Further analysis using a multi-locus phylogeny and additional samples from the Sundaic region are required to clarify the broad-scale diversification and biogeographical pattern of SE-Asian *Macrobrachium* fauna.

## Conclusion

This study provides the first DNA barcode library and cryptic evidence of genus *Macrobrachium* in Indochina. Diagnostic characters of some species have been detected from nucleotide positions and can be included as additional characters for taxonomic identification and species validation (S1 Table). Despite wide geographical dispersion, several species show low genetic affinity between different geographical populations. In contrast, the morphologically complex *Macrobrachium* species group possesses high genetic diversity and the geographical distribution shows allopatry between Chao Phraya and Mekong river basins. The broad scale phylogenetic relationships of Indochinese species from the COI dataset are still unresolved. However, examples of stable clade composition and monophyletic lineages are found, especially in estuarine species. The DNA species delimitation suggests several candidate OTUs which might be cryptic species hidden within common species, such as the *M*. *lanchesteri*, *M*. *sintangense* and *M*. *pilimanus* species groups. The barcode gap analysis provides a delimitation threshold for Indochinese taxa despite the high intraspecific variation detected in some species. The inappropriate taxonomic identification of some available sequences from the public database raises caution and suggests that dataset reconstruction and re-verification for further taxonomic comparison is required. Finally, the results of this study indicate that the regional fauna share interconnection with other neighboring regions such as India-Burma and East Asia, as indicated by the records of some widely dispersed species.

## Supporting information

S1 Table Nucleotide diagnostic characters of Indochinese *Macrobrachium* species. (XLSX)

S1 Fig ML and BI phylogenetic trees of Indochinese *Macrobrachium* with statistical node support. (ZIP)
