# Supplementary figures and images for: Evidence of cryptic diversity in freshwater Macrobrachium prawns from Indochinese riverine systems revealed by DNA barcode, species delimitation and phylogenetic approaches
Source: PLoS One. 2021 Jun 2;16(6):e0252546. doi: 10.1371/journal.pone.0252546 (PMC8171930; doi:10.1371/journal.pone.0252546)

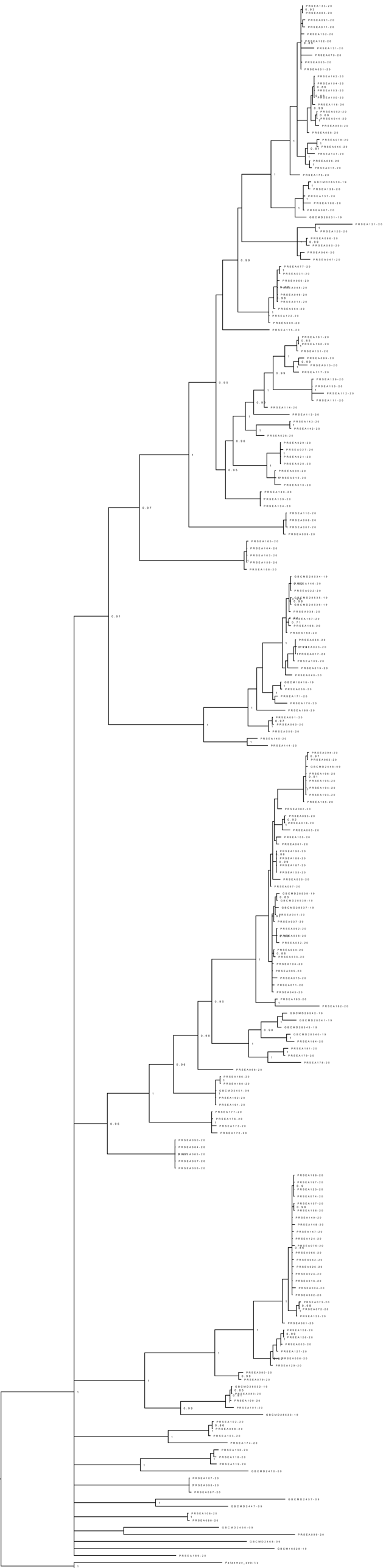

Supplement: S1 Fig — (ZIP) [file pone.0252546.s002.zip › Fig S1/Fig S1 BI.pdf]

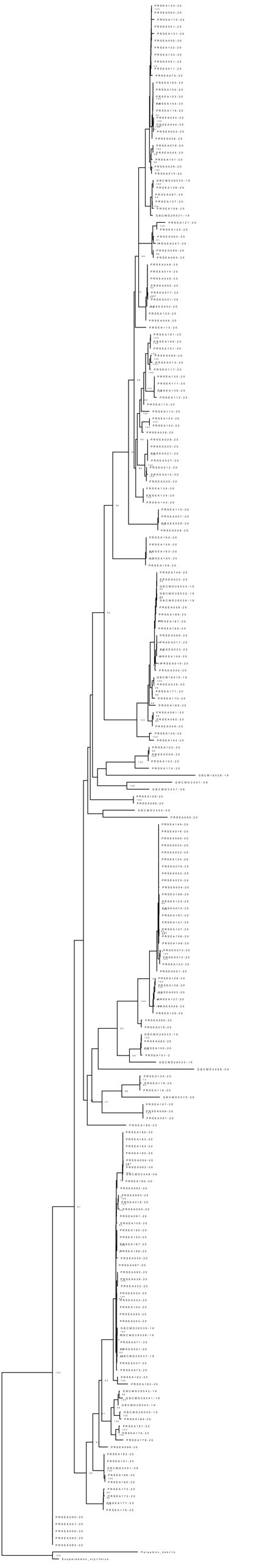

Supplement: S1 Fig — (ZIP) [file pone.0252546.s002.zip › Fig S1/Fig S1 ML IQ.pdf]

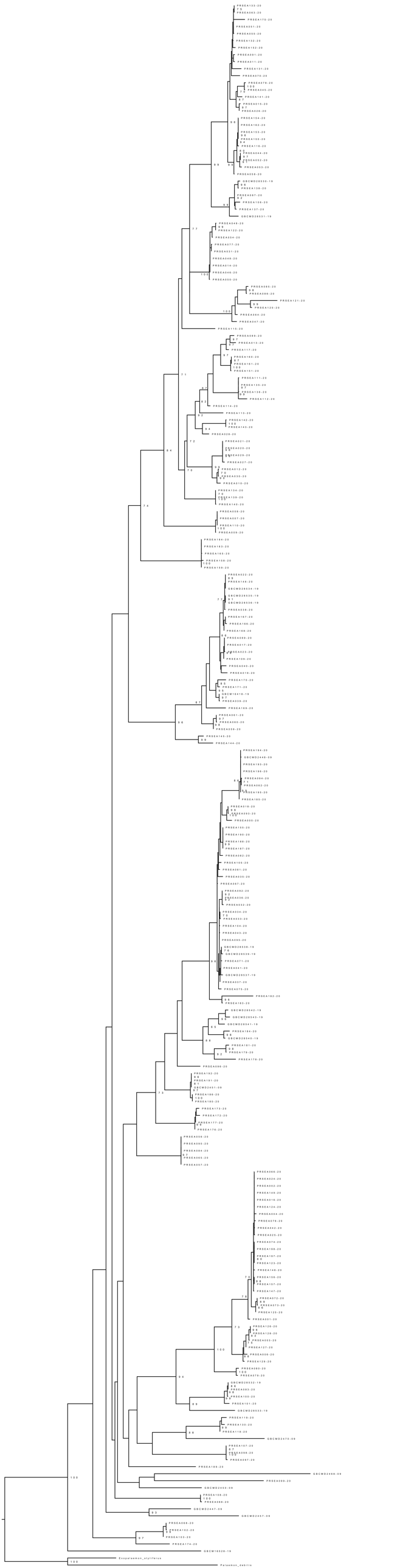

Supplement: S1 Fig — (ZIP) [file pone.0252546.s002.zip › Fig S1/Fig S1 ML RAxML.pdf]
